# Conformational Changes of Blood ACE in Chronic Uremia

**DOI:** 10.1371/journal.pone.0049290

**Published:** 2012-11-16

**Authors:** Maxim N. Petrov, Valery Y. Shilo, Alexandr V. Tarasov, David E. Schwartz, Joe G. N. Garcia, Olga A. Kost, Sergei M. Danilov

**Affiliations:** 1 Department of Chemistry, Lomonosov Moscow State University, Moscow, Russia; 2 Department of Nephrology, Moscow University for Medicine and Dentistry, Moscow, Russia; 3 Department of Anesthesiology, University of Illinois at Chicago, Chicago, Illinois, United States of America; 4 Institute for Personalized Respiratory Medicine, University of Illinois at Chicago, Chicago, Illinois, United States of America; 5 National Cardiology Research Center, Moscow, Russia; Federal University of São Paulo (UNIFESP), Escola Paulista de Medicina, Brazil

## Abstract

**Background:**

The pattern of binding of monoclonal antibodies (mAbs) to 16 epitopes on human angiotensin I-converting enzyme (ACE) comprise a conformational ACE fingerprint and is a sensitive marker of subtle protein conformational changes.

**Hypothesis:**

Toxic substances in the blood of patients with uremia due to End Stage Renal Disease (ESRD) can induce local conformational changes in the ACE protein globule and alter the efficacy of ACE inhibitors.

**Methodology/Principal Findings:**

The recognition of ACE by 16 mAbs to the epitopes on the N and C domains of ACE was estimated using an immune-capture enzymatic plate precipitation assay. The precipitation pattern of blood ACE by a set of mAbs was substantially influenced by the presence of ACE inhibitors with the most dramatic local conformational change noted in the N-domain region recognized by mAb 1G12. The “short” ACE inhibitor enalaprilat (tripeptide analog) and “long” inhibitor teprotide (nonapeptide) produced strikingly different mAb 1G12 binding with enalaprilat strongly increasing mAb 1G12 binding and teprotide decreasing binding. Reduction in S-S bonds via glutathione and dithiothreitol treatment increased 1G12 binding to blood ACE in a manner comparable to enalaprilat. Some patients with uremia due to ESRD exhibited significantly increased mAb 1G12 binding to blood ACE and increased ACE activity towards angiotensin I accompanied by reduced ACE inhibition by inhibitory mAbs and ACE inhibitors.

**Conclusions/Significance:**

The estimation of relative mAb 1G12 binding to blood ACE detects a subpopulation of ESRD patients with conformationally changed ACE, which activity is less suppressible by ACE inhibitors. This parameter may potentially serve as a biomarker for those patients who may need higher concentrations of ACE inhibitors upon anti-hypertensive therapy.

## Introduction

Angiotensin I-converting enzyme (ACE, CD143, EC 3.4.15.1), a zinc-metallopeptidase, is a key regulator of blood pressure participating in the development of vascular pathology and remodeling [1]–[3]. The somatic isoform of ACE (sACE) is highly expressed as a type-I transmembrane glycoprotein in endothelia [4]–[7], epithelia and neuroepithelia [8]–[10], as well as immune cells – macrophages and dendritic cells [11]–[12]. ACE has been designated as a CD marker – CD143 [13]–[14]. Somatic ACE also presents as a soluble form, for example, in plasma, cerebrospinal and seminal fluids, that lacks the transmembrane domain responsible for membrane attachment [15].

In healthy individuals, the level of ACE in the blood is very stable [16], whereas significant increase (2-4-fold) in blood ACE activity was observed in granulomatous diseases such as sarcoidosis and Gaucher’s disease [15], [17]–[20]. Less dramatic, but still significant increase in blood ACE activity was reported in patients with renal diseases and at uremia [21]–[23].

Under normal conditions, serum ACE likely originates from ACE released from endothelial cells [24], perhaps, mainly lung capillaries [7] by proteolytic cleavage by still unidentified membrane-bound secretase [25].

Two homologous domains (N and C domains) within a single polypeptide chain comprise the majority of the structure of sACE, each containing a functional active center [26]. The three-dimensional crystal structure of sACE is still unknown. However, the models of the two-domain ACE has been recently suggested [27]–[29], based on the solved crystal structures of the C and N domains [30]–[31], epitope mapping of monoclonal antibodies (mAbs) to ACE [27], and on the electron microscopy picture of sACE [28].

To provide structure-function information on ACE molecule, we previously developed a set of ∼40 mAbs directed to sequential and conformational epitopes to human, rat and mouse ACE [27], [32]–[36], which proved useful for ACE quantification in solution by ELISA [37] and by flow cytometry [12], [38]. These mAbs have facilitated the investigation of the structure and function of ACE [27], [32], [39]–[45] and were successfully used for the detection of carriers of novel ACE gene mutations such as Pro1199Leu [46], Trp1197Stop [47], Gln1069Arg [48], and Tyr465Asp [29].

Recent ACE studies with mAbs recognizing different conformational epitopes on the surface of the catalytically active N domain (eight mAbs) and the C domain (eight mAbs) of human ACE molecule revealed that the pattern of mAb binding to ACE is potentially a very sensitive marker of the local conformation of ACE globule. The changes of this pattern could be definitely attributed to the changes of the epitopes for the distinct mAbs due to denaturation of ACE globule, chemical modification, inhibitor binding, mutations, and different glycosylation/deglycosylation [49].

**Figure 1:**
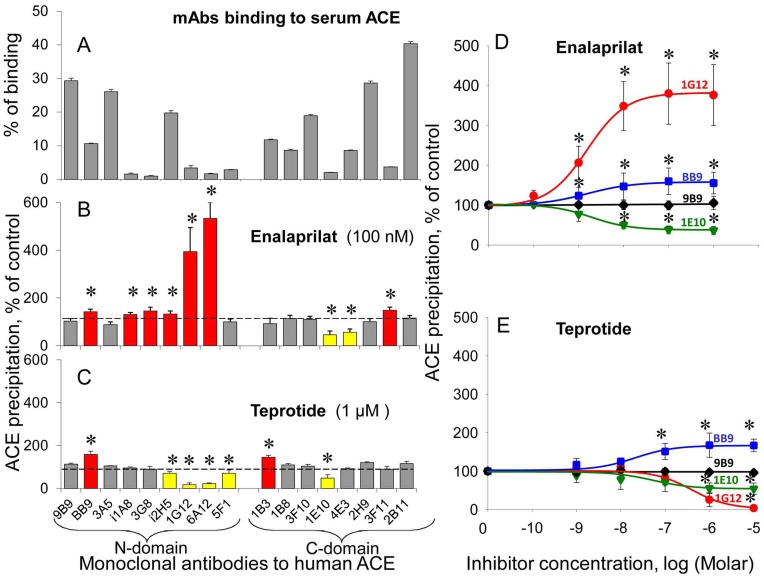
Effect of ACE inhibitors on the binding of a set of anti-ACE mAbs to blood ACE. The binding of a set of 17 mAbs to the N and C domains of human sACE to the human serum ACE was determined using plate precipitation assay [32], [49] with ZPHL as a substrate. Serum pooled from 30 healthy donors and diluted 1/5 in PBS (50 µl) was incubated with or without ACE inhibitors for 1 hour at 37oC and then was incubated overnight with microtiter plate coated with different anti-ACE mAbs (3 µg/ml) via goat-anti-mouse bridge. Serum components, inhibitors, as well as unbound ACE, were then eliminated by washing. ACE activity precipitated by each of tested mAbs was determined by adding ZPHL solution (in 100 mM potassium phosphate buffer, containing 300 mM KCl, 1 µM ZnSO4, pH 8.3) directly into the wells. After 1–4 hours at 37°C the product of the enzymatic reaction, His-Leu, was quantified by reaction with o-phthaldialdehyde spectrofluorometrically directly in the wells. **A**. Comparative binding of the mAbs set to serum ACE. **B–C**. Data are expressed as a percentage of precipitated ACE activity from serum pre-incubated with enalaprilat (100 nM - **B**) or teprotide (1 µM - **C**) by a given mAb from that w/o inhibitor. The colored columns show antibodies which binding to blood ACE in the presence of ACE inhibitor differed (more than 20%) from that to without the inhibitor: mAbs increasing or decreasing their binding to ACE in the presence of inhibitor are marked by red or yellow, respectively. **D–E**. Effect of different concentrations of enalaprilat (**D**) or teprotide (**E**) on mAbs binding to serum ACE. Data are mean ± SD of 3–8 independent experiments (each in duplicates). *, p<0.05 in comparison with mean values for samples without ACE inhibitors.

Based on these systematic studies of ACE epitopes [27], [32], [42]–[45], [49]–[50], we hypothesized that the pattern of precipitation of ACE activity by this set of mAbs, i.e. the “conformational fingerprinting of ACE”, may detect conformationally changed ACE in the blood as a result of a disease. Uremia is characterized by an elevated level of toxic compounds [51] and therefore served as a disorder of interest for assessing blood ACE conformation.

**Figure 2:**
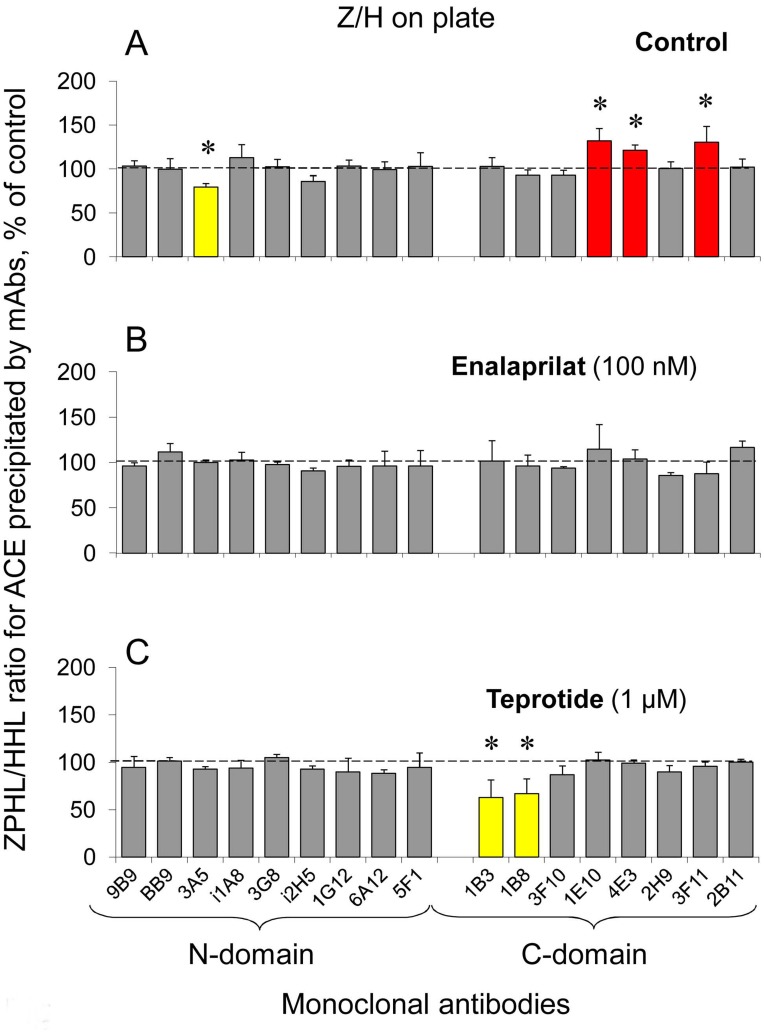
Effect of ACE inhibitors on the comparative rates of ZPHL and HHL hydrolysis by blood ACE bound by mAbs on the plate. **A**. ACE activity precipitated from citrated pooled plasma by each given mAb was determined spectrofluorometrically directly in the wells (as in Fig. 1) with two substrates for ACE, HHL and ZPHL. Data are expressed as the ratio of the rates of the hydrolysis of two substrates (ZPHL/HHL ratio) by ACE bound by mAbs in comparison with the mean value obtained for all 17 mAbs. * - p<0.05 in comparison with mean value for the whole set of mAbs. **B–C**. Effect of enalaprilat (100 nM – **B**) and teprotide (1 µM – **C**) on ZPHL/HHL ratio determined for ACE bound with each mAb. * - p<0.05 in comparison with mean values for samples without ACE inhibitors. All other terms and conditions are as in Fig. 1. Red colored columns show those mAbs which binding with ACE increased (and yellow – decreased) ZPHL/HHL ratio. Data are mean ± SD from 3–8 independent experiments (each in duplicates). p<0.05 in comparison with mean values for samples without ACE inhibitors.

Here we report the findings which support this hypothesis and suggest that ACE “conformational fingerprinting” provides a potential tool for the future selection of a subgroup of ESRD patients, whose ACE inhibitor therapy, if needed, should be more aggressive in order to be effective.

**Figure 3:**
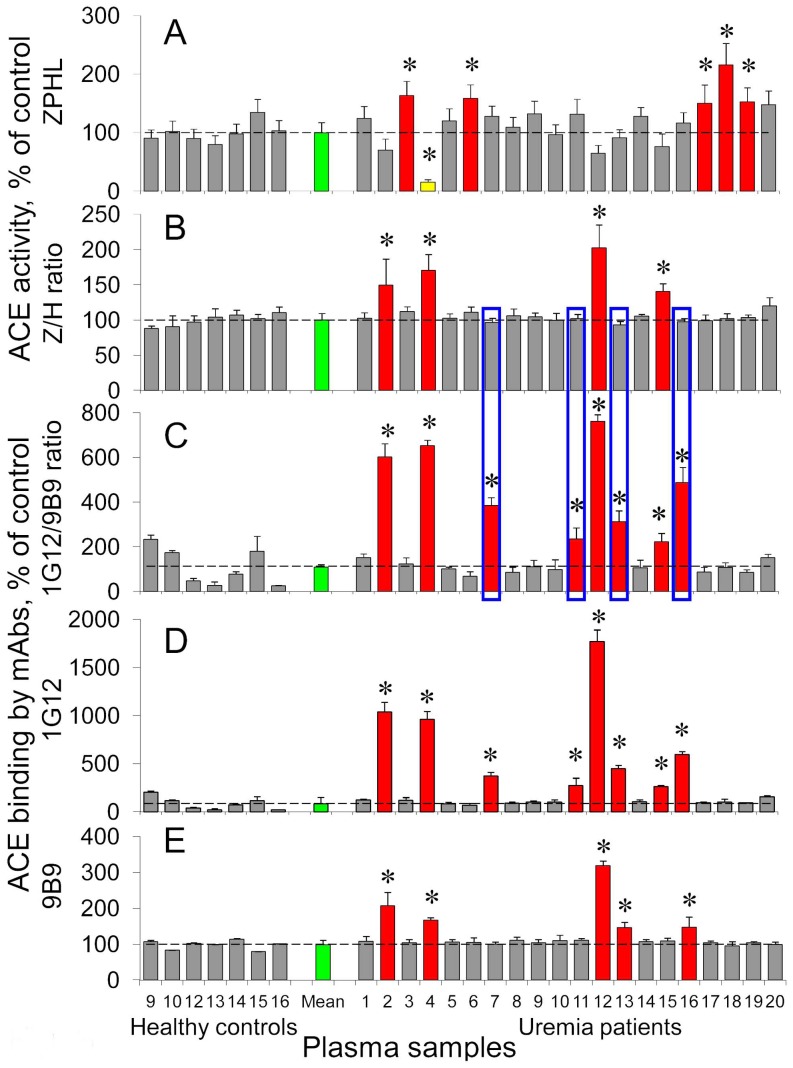
Blood ACE phenotyping in uremia. **A**. ACE activity in the citrated plasma (diluted 5-fold) of patients with uremia (versus that of healthy volunteers) was determined spectrofluorometrically using 20 µl of diluted plasma with 100 µl of ACE substrate ZPHL at 1 hour of incubation with substrate. B. **The ratio of the rates of the hydrolysis of two substrates (ZPHL/HHL ratio) for ACE from the blood of patients with uremia and from the blood of healthy persons. C-E**. ACE activity precipitated by different mAbs from citrated plasma of uremic patients (versus that from plasma of healthy donors: **E** - by mAb 9B9; **D** - by mAb 1G12 and **C** – their ratio for each plasma sample. Results are shown as mean ± SD from 3 to 10 independent experiments, each in duplicates or triplicates. The red color of the columns shows plasma of those patients whose blood parameter (ACE activity, Z/H ratio, 1G12/9B9 ratio/mAb 9B9 or 1G12 binding) was significantly higher (and yellow bar-significantly lower) than mean value for healthy donors. The green bars shows mean values for healthy controls. Boxes show those patients which have increased 1G12/9B9 ratio while normal ZPHL/HHL ratio. All other terms and conditions are as in Fig. 1. * - p<0.05 in comparison with mean value for healthy controls.

**Figure 4:**
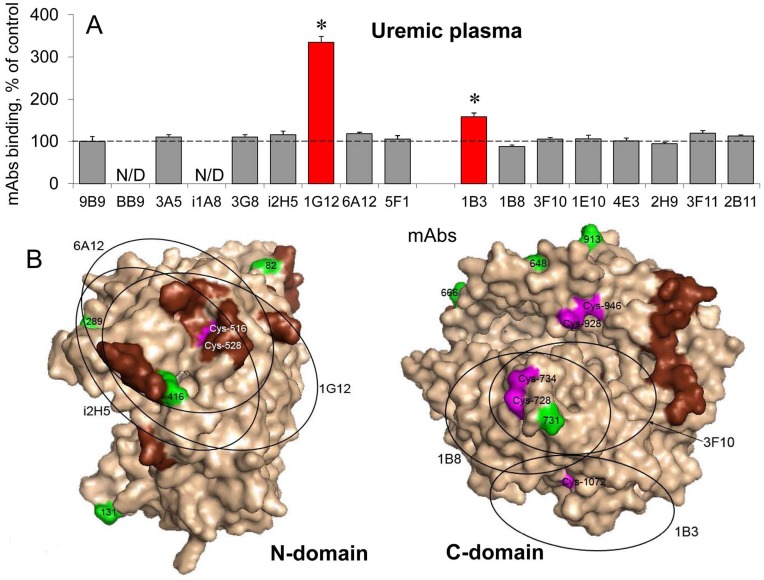
Conformational fingerprinting of uremic plasma with highly elevated 1G12/9B9 ratio and normal Z/H ratio. **A**. ACE activity precipitated by different mAbs from plasma of uremic patients with highly elevated 1G12/9B9 ratio and normal Z/H ratio (mean data for uremic samples #7 and #16 from Fig. 3) as a percentage from that for plasma from uremic persons with normal 1G12/9B9 and ZPHL/HHL ratios (mean data of two uremic samples #5 and #18 from Fig. 3) taken as control. Data presented are mean ± SD of duplicates from 2 experiments. The red-colored columns show those antibodies which binding to blood ACE in uremic patients with high 1G12/9B9 ratio differ more than 20% from that for patients with normal 1G12/9B9 ratio. * – parameter from the test sample was statistically different (p<0.05) from the healthy control. **B**. Localization of disulfide bridges and free cysteine residues in the epitopes for mAbs 1G12 together with overlapping epitopes for mAbs 6A12 and i2H5 on the N domain of ACE (left) and mAb 1B3 together with adjacent epitopes for mAbs 1B8 and 3F10 (on the C domain of ACE ). The regions of the epitopes for different mAbs were marked by circles with diameter of approximately 30Å, which corresponds to the square of 600–900 Å^2^. Cysteine residues are marked magenta; potential glycosylation sites are marked green; amino acid residues participating in hinge-bending movement of domains are marked brown.

## Experimental Section

### ACE Activity Assay

ACE activity in citrate plasma, serum, cell lysates or culture medium was measured by fluorimetric assay [52]–[53]. Briefly, 20–40 µl aliquots of ACE source, diluted correspondingly in PBS-BSA (0.1 mg/ml), were added to 200 µl of ACE substrate (5 mM Hip-His-Leu or 2 mM Z-Phe-His-Leu) in phosphate buffer, pH 8.3 (with 300 mM NaCl) and incubated for the appropriate time at 37°C. His-Leu product was quantified with *o*-phthaldialdehyde spectrofluorometrically (365 nm excitation and 500 nm emission wavelengths). Determination of the ratio of the rates of the hydrolysis of two substrates (ZPHL/HHL) was performed as described [54]. ACE activity in serum/plasma was also determined with 0.3 mM angiotensin I as a substrate as described above.

**Figure 5:**
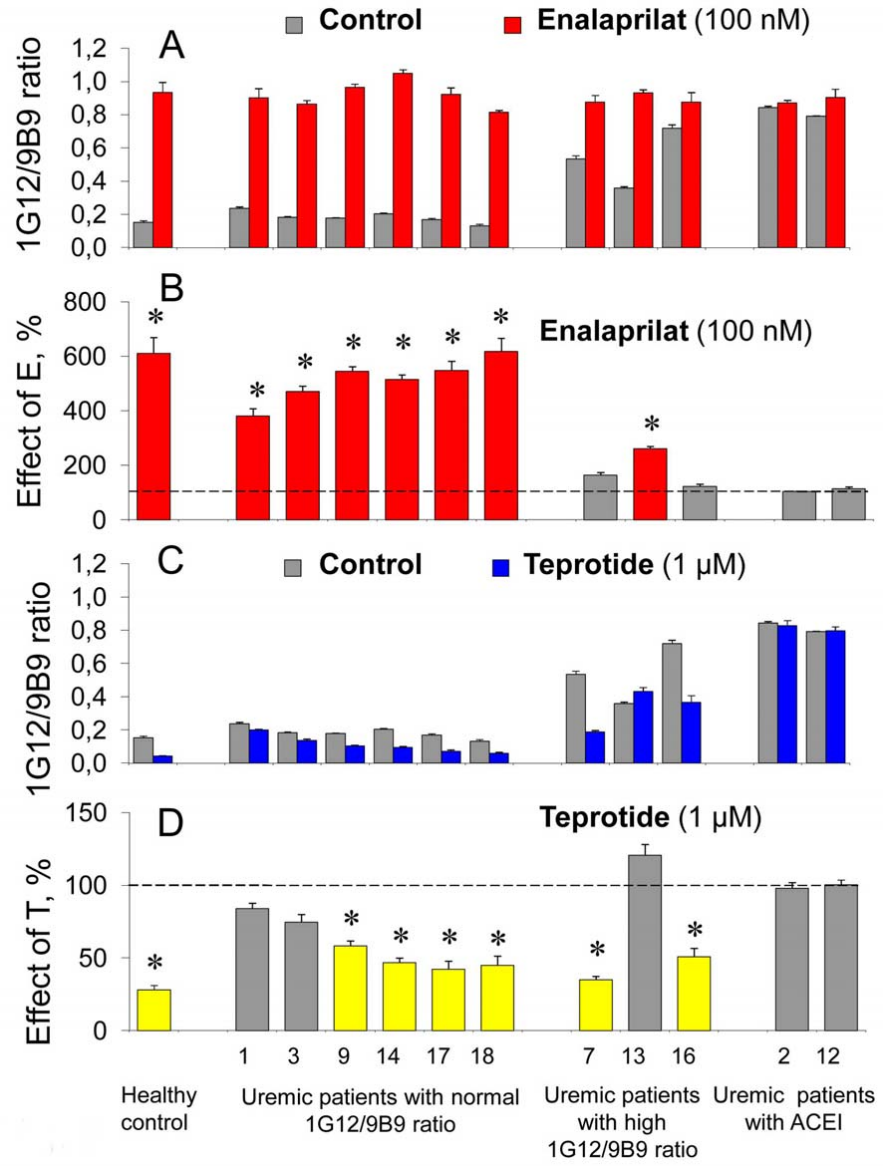
Effect of ACE inhibitors on the local conformation of blood ACE in uremia. **A**. The ratio of ACE activities precipitated from the citrated plasma of uremic patients by mAbs 1G12 and 9B9 (1G12/9B9 ratio) and from pooled plasma from healthy volunteers in the presence of enalaprilat (100 nM) – red bars and without the inhibitor – grey bars. **B**. The data presented on Fig. 5A were also expressed as a percentage of the effect of enalaprilat on the values of 1G12/9B9 ratio for different patients. The red columns show those mAbs for which enalaprilat effect (increase of mAb binding) exceeded 20%. **C**. The ratio of ACE activities precipitated from the citrated plasma of uremic patients by mAbs 1G12 and 9B9 (1G12/9B9 ratio) and from pooled plasma from healthy volunteers in the presence of teprotide (1 µM) – blue bars and without the inhibitor – grey bars. **D**. The data presented on Fig. 5C were also expressed as a percentage of the effect of teprotide on the values of 1G12/9B9 ratio for different patients. The yellow columns show those mAbs for which teprotide effect (decrease of mAb binding) exceeded 20%. All other terms and conditions – as in Figure 1. Data are mean ± SD of 3 independent experiments (each in duplicates). *, p<0.05 in comparison with mean value obtained without inhibitor.

**Figure 6:**
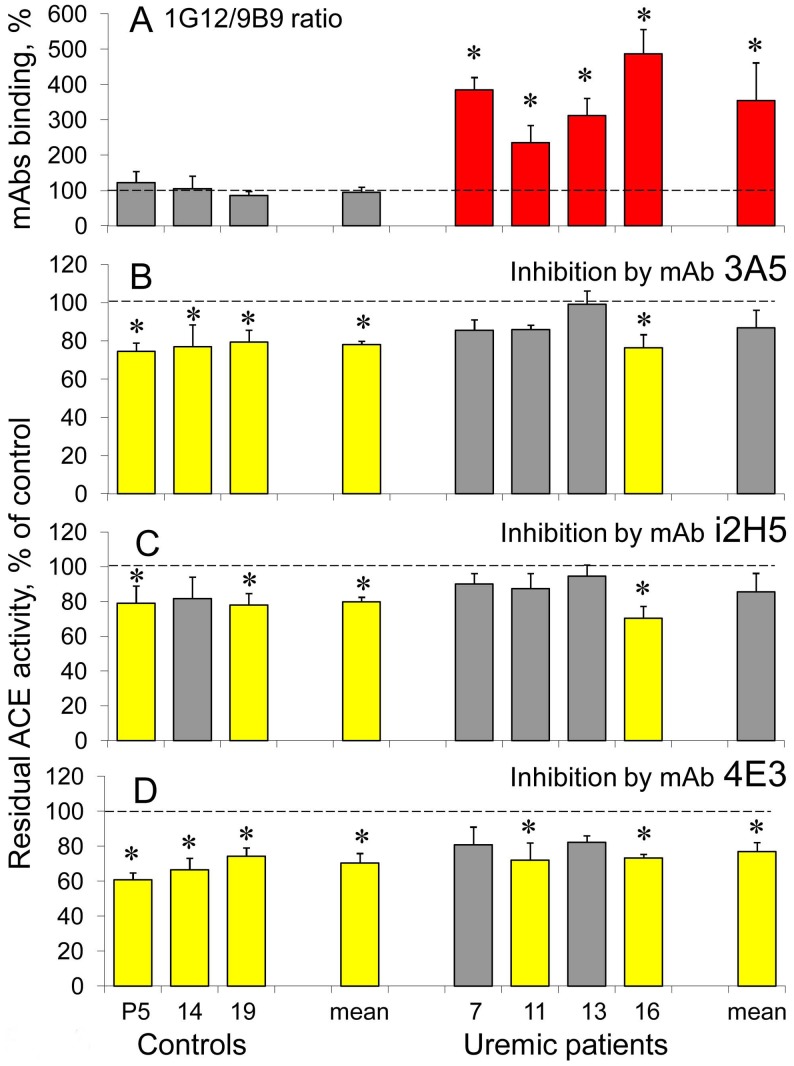
Effect of anti-catalytic mAbs on plasma ACE in uremia. **A**. The ratio of ACE activities precipitated from the citrated plasma of uremic patients with high 1G12/9B9 ratio and normal ZPHL/HHL ratio and from healthy controls (P5– pooled citrated plasma, #14 and #19– individual plasmas) by mAbs 1G12 and 9B9 (1G12/9B9 ratio. Grey bars – healthy controls; red bars – uremic patients with high 1G12/9B9 ratio with p<0.05 in comparison with mean value. **B-D**. Effect of anti-catalytic mAbs on ACE activity. The effects of anti-N domain mAbs 3A5 and i2H5 [42] were determined with ZPHL as a substrate. The effect of anti-C domain mAb 4E3 [27] was determined with HHL as a substrate. Data are presented as a residual ACE activity after incubation of tested mAbs (10 µg/ml) with citrated plasma (diluted 5-fold). Grey bars – ACE inhibition less than 20%, yellow bars – more than 20% of ACE inhibition. Data are mean ± SD of 3 independent experiments (each in duplicates), with p<0.05 in comparison with mean value obtained without anti-catalytic mAb.

**Figure 7:**
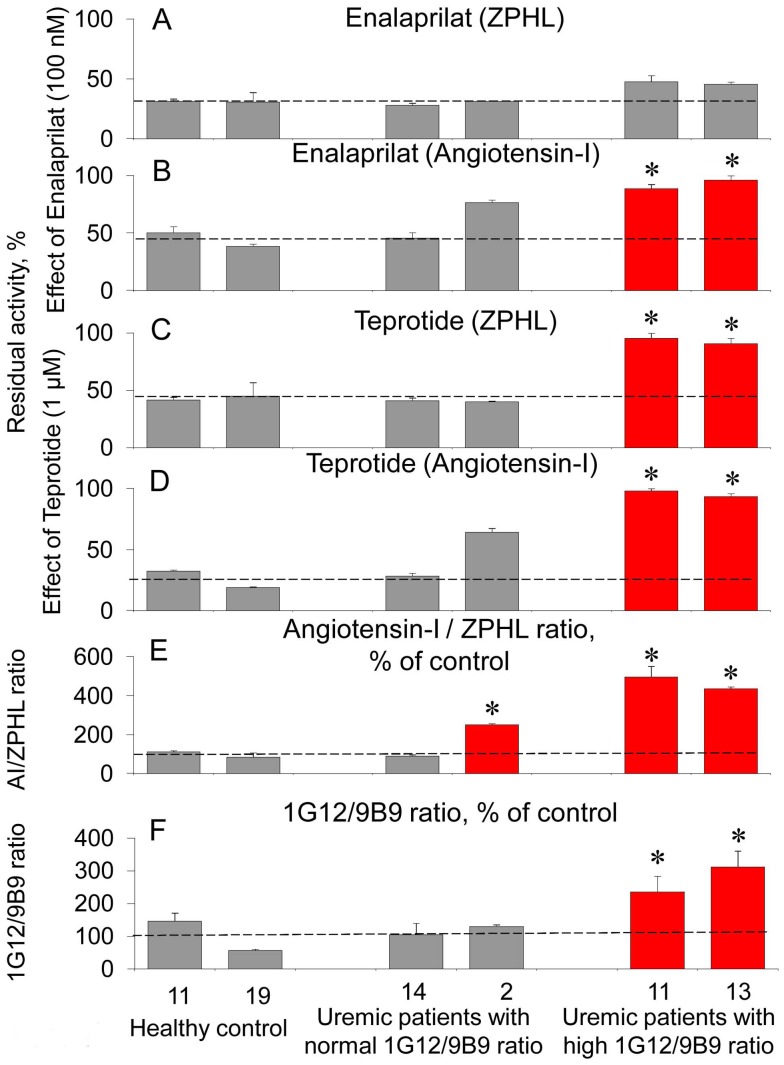
Effect of ACE inhibitors on the activity of plasma ACE in uremia. **A**–**D** Citrated plasma samples of uremic patients with normal (patients #14 and #2) and high (patients #11 and #13) 1G12/9B9 ratio (versus healthy controls, #11 and #19) were incubated with “short” ACE inhibitor enalaprilat (100 nM, **A** and **B**) and “long” ACE inhibitor teprotide (1 µM, **C** and **D**) for 1 hour. Data are presented as a residual ACE activity determined with “short” substrate ZPHL (0.5 mM, **A** and **C**) and “long” substrate angiotensin I (0.3 mM, **B** and **D**). Data are presented as a residual ACE activity. **E**. The ratio of the rates of the hydrolysis of angiotensin I and ZPHL (angiotensin I/ZPHL ratio) for corresponding samples. **F**. mAb1G12/9B9 binding ratio for corresponding samples expressed as % from the mean value for healthy persons. Grey bars – inhibition of ACE activity (A–D) or parameters measured in E–F in uremic samples was not differed from that for healthy patients with low 1G12/9B9 ratio. Red bars –measured parameters were statistically higher than that in healthy patients with low (normal) 1G12/9B9 ratio *, p<0.05 in comparison with mean value for healthy patients. Data are mean ± SD from 3 independent experiments (each in duplicates).

**Figure 8:**
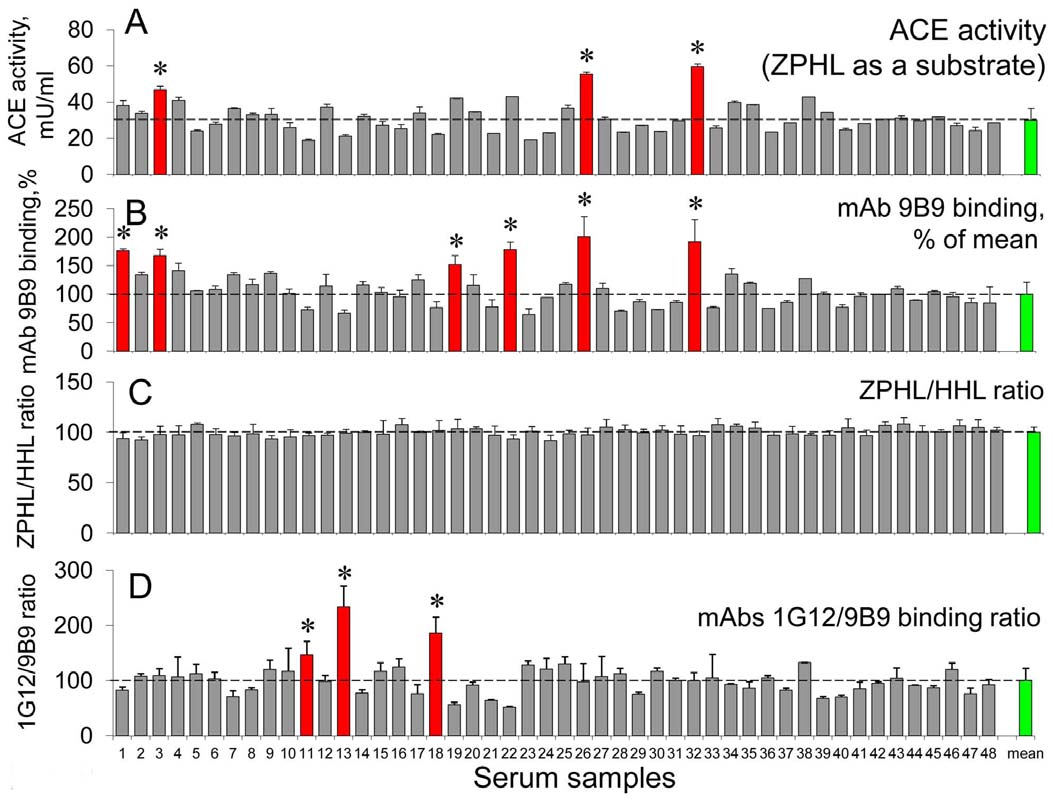
Blood ACE phenotyping in healthy donors. **A**. ACE activity in serum of healthy young donors was determined as in Figure 3. B. **ACE activity precipitated from serum of healthy donors by mAb 9B9 (estimation of relative ACE protein content).** C. **The ratio of the rates of the hydrolysis of two substrates (ZPHL/HHL ratio) for tested serum samples was determined as in** **Figure 3** **. D**. ACE activity precipitated from serum of healthy donors by mAbs 9B9 and 1G12 and expressed as their ratio for each serum sample. The red color of the bar shows serum of those patients whose tested parameters were significantly higher (and yellow bar – significantly lower) than mean value for all samples (green bars). Results are shown as mean ±SD of 4 independent experiments, each in duplicates or triplicates. *, statistically significant difference (p<0.05) from the mean value.

### Isolation and Cultivation of ACE-expressing Cells

Plasmids carrying the coding sequence for wild-type (WT) ACE (based on pcDNA3.1, Invitrogen Corp., Carlsbad, CA) containing the full-length somatic ACE cDNA controlled by CMV early promoter [55] or mutant ACEs – i) WTΔ –without transmembrane anchor [56], kindly provided by Dr. F. Alhenc-Gelas (then INSERM Unit 352, Paris, France); ii) truncated N domain - D629 [57]; iii) testicular ACE –tACE [58], kindly provided by Dr. E. Sturrock (University of Cape Town, South Africa) were stably expressed in CHO cells (ATCC, Manassas, VA) using Plus Reagent (Invitrogen Corp., Carlsbad, CA) as described [55]. Stable transfected cells expressing ACE at confluence were washed gently with PBS and incubated 24–48 hours with “complete culture medium” (Mediatech, Inc, Herndon, VA) or Ultra-CHO medium (Cambrex Bio-Science Walkersville, Inc, Walkersville, MD) without fetal bovine serum (FBS). Culture medium was collected as a source of soluble ACE**.** Cell lysates were used as a source of a membrane form of wild-type and mutant ACEs for biochemical and immunological characterization. After 24 hours of the culturing the culture medium was aspirated and centrifuged (to precipitate possible detached cells) and the cells, after washing by PBS, were lysed with 50 mM Tris-HCl buffer, pH 7.5, containing 150 mM NaCl and 0.5% Triton X-100. ACE activity in the lysates and culture medium was determined with two ACE substrates as described above.

**Table 1:** Clinical Data.

1^st^ GroupConformationally naïve ACE					2^nd^ GroupConformationally changed ACE					3^rd^ GroupPresence of ACE inhibitors				
Patient##	Age,Years	DV^1^months	SBP^2^mm,Hg	DBP^3^mm,Hg	Patient##	Age,Years	DV^1^months	SBP^2^mm,Hg	DBP^3^mm,Hg	Patient##	Age,Years	DV^1^months	SBP^2^mm,Hg	DBP^3^mm,Hg
1	47	147	137	80	7	58	119	156	72	2	43	216	140	90
3	75	161	72	42	11	61	216	158	90	4	65	160	130	80
5	48	190	130	75	13	61	126	130	70	12	57	184	138	77
6	48	98	140	78	16	47	115	137	80	15	63	125	80	45
8	53	228	123	70										
9	39	137	97	56										
10	72	177	162	80										
14	62	120	129	74										
17	58	109	110	60										
18	72	141	140	66										
19	63	128	87	53										
20	73	123	160	60										
Mean	**59.2**	**146.6**	**123.9**	**66.2**		**56.8**	**144.0**	**145.3**	**78.0**		**57.0**	**171.3**	**122.0**	**73.0**
SD	12.2	37.1	27.8	12.1		6.7	48.2	13.9	9.1		9.9	38.4	28.3	19.5
p value	For difference with 1^st^ group					0.629	0.926	0.069	0.078		0.217	0.06	0.974	0.547
p value	For difference with 2^nd^ group										0.968	0.411	0.191	0.659

1 DV- Dialysis Vintage; ^2^SBP- Systolic Blood Pressure; ^3^DBP- Diastolic Blood Pressure.

Blood pressure was measured before dialysis and before blood sampling.

### Immunological Characterization of ACE from Different Sources (Plate Immunoprecipitation Assay)

Plastic 96-well plates (Corning, Corning, NY) were coated with 50 µl of 10 µg/ml affinity-purified goat anti-mouse IgG (Pierce, Rockford, IL) and stored overnight at 4°C. After washing with PBS/0.05% Tween 20, the wells were incubated with different anti-ACE mAbs (2 µg/ml) in PBS/BSA (0.1 mg/ml) for 2 hrs at RT and washed. mAb BB9 to the N domain of ACE [59] which has an epitope overlapping with epitopes of 9B9 and 3A5 (Danilov et al., unpublished observation) was kindly provided by Dr. P. Simmons (Brown Foundation Institute of Molecular Medicine (IMM), University of Texas Health Science Center, Houston, TX). Wells were then incubated with 50 µl of ACE from any source (plasma/serum from volunteers or patients, soluble ACE secreted from cells transfected with wild type or mutant ACEs, lysate from these cells, or ACE purified from serum or seminal fluid by affinity chromatography on lisinopril-Sepharose as in [10]). Some samples (e.g., soluble ACE versus membrane-bound ACE) were preliminarily equilibrated by ACE activity with Z-Phe-His-Leu as a substrate. After washing of unbound ACE, plate-bound ACE activity was measured by adding a substrate (Hip-His-Leu or Z-Phe-His-Leu) directly into the wells [49].

In some experiments, the source of ACE (pure ACE from serum or seminal fluid, plasma/serum, seminal fluid, soluble or membrane form of recombinant ACE) was preliminarily incubated with the effectors (ACE inhibitors, glutathione, dithiotreitol, etc.) and then added to the 96-well plate covered by different anti-ACE mAbs. Hemolysis of erythrocytes was performed as follows: several drops of distilled water were added to the whole blood (9 ml), the blood was vigorously shaken for 10 minutes, incubated at 4° for 14 hours and then centrifuged to obtain hemolysed serum.

### Analysis of ACE Activity in Blood from Healthy Volunteers and Uremic Patients

This non-interventional pilot study was approved by the Institutional Review Boards of Moscow State University, clinical base of Moscow University for Medicine and Dentistry - Euromedic International Dialysis Centers, and University of Illinois at Chicago, and the procedures followed were in accordance with institutional guidelines. After providing informed consent regarding blood samples and data processing, citrated plasma or serum was obtained from healthy donors, from 20 patients with End Stage Renal Disease (ERSD) on maintenance hemodialysis (HD) or from age-match control patients without kidney diseases. Samples were kept at −18° not longer than 3 months until further determination of ACE activity and immunochemical characterization of ACE. All ESRD patients were on maintenance HD with standard dialysis prescription (4 h × 3 times per week schedule), mean age of patients (mean±SD) was 58.3±8.5 years, mean dialysis vintage was 151±32 months. Hemodialysis procedures were performed on Fresenius 4008S dialysis machines with low flux polysulfone dialyzers F-series. All patients match major dialysis adequacy criteria - Urea Reduction Rate (URR)>65%, Gotch dialysis index (KT/V)>1.4 and maintain stable Hb level from 10 to 12 g/dl on low dose epoetin beta and i.v. iron (iron sucrose) preparations. Concomitant therapy included calcium salt as a phosphate binder, active vitamin D and/or cinacalcet in some patients with secondary hyperparathyreosis. Though all patients had a past history of severe hypertension, some patients continued to intake antihypertensive medications at the time of the study - ACE inhibitors, AT2 receptor blockers (ARBs), calcium channel blockers (CCBs) or beta blockers.

## Results and Discussion

### Effect of ACE Inhibitors on ACE Conformational Fingerprinting

The presence of toxic compounds in the blood could influence the pattern of mAbs binding to blood ACE. Thus, in the course of mAbs 1G12 and 6A12 (to the N domain of ACE) epitope mapping we determined that the binding of these mAbs to blood ACE increased dramatically in the presence of common ACE inhibitors (captopril, lisinopril, enalaprilat), even facilitating the development of a sensitive assay for detection/quantification of ACE inhibitors in blood [43].

We performed a study of ACE inhibitor effects on the binding of a panel of 17 mAbs to blood ACE (“conformational fingerprinting” approach) revealed by ACE activity precipitated by each mAb. The relative binding of strong mAbs (2B11, 9B9, 3A5, 2H9) dramatically differed compared to weak mAbs (i1A8, 3G8, 1E10, 3F11), likely reflecting difference in affinity constants for these mAbs (Fig. 1A). Figure 1B demonstrates that in addition to dramatic effect of enalaprilat on the binding of mAbs 1G12 and 6A12 [43] this commercially available and commonly used “short” (tripeptide analog) ACE inhibitor weakly (but significantly) increased precipitation of ACE activity with 5 other mAbs - 4 to the N domain (BB9, i1A8, 3G8, i2H5) and 1 to the C domain (3F11) but decreased measured precipitated ACE activity with two mAbs - 1E10 and 4E3 to the C domain. The colored columns show those antibodies which binding to serum ACE in the presence of enalaprilat differ from that without ACE inhibitor.

Interestingly, the effect of the “long” (nonapeptide) ACE inhibitor teprotide (BPP9a, Glp-Trp-Pro-Arg-Pro-Gln-Ile-Pro-Pro) on the precipitation of serum ACE activity was different - teprotide increased mAb BB9 and decreased mAb 1E10 binding to ACE similar to enalaprilat but dramatically decreased the binding of mAbs 1G12 and 6A12 (with partially overlapping epitopes) as opposite to enalaprilat (Figure 1C). The effects of both inhibitors on mAbs binding to ACE were confirmed at a wide range of their concentrations (Figures 1D and 1E), thus demonstrating that the binding of the inhibitors with different structures within ACE active centers causes strikingly different conformational changes of the surface of ACE globule.

The increase in precipitated ACE activity in the presence of ACE inhibitor (most visibly seen in the case of mAbs 1G12/6A12 and enalaprilat, Figures 1B and 1D) could be only due to the increase in ACE binding by a particular mAb. However, when ACE activity precipitated by any mAb decreased in the presence of inhibitor the situation was not so simple. The decrease in ACE activity precipitated by any particular mAb and measured directly in the well could be attributed either to a decrease of the efficiency of ACE binding with this mAb or just to some retention of ACE inhibitor within ACE active centers induced by mAb binding.

In order to clarify this point we used previously described approach that the relative rate of the hydrolysis of two ACE substrates Z-Phe-His-Leu (ZPHL) and Hip-His-Leu (HHL) can indicate the presence of ACE inhibitors in the reaction media as the ratio ZPHL/HHL dramatically increased in the presence of inhibitors [54]. Fig. 2A shows that ZPHL/HHL ratio for blood ACE (without any additional inhibitor) precipitated by most mAbs did not differ from the corresponding value obtained for blood ACE in solution. This ratio was lower for ACE precipitated by anti-catalytic mAbs for the N domain, 3A5 and i2H5, due to selective inhibition by these mAbs of the N domain [32], [42] and was higher in the case of anti-catalytic mAbs for the C domain, 1E10 and 4E3, due to selective inhibition of the C domain [27], similarly to the effect of these mAbs on ZPHL/HHL ratio for ACE in solution [54] (and Fig. S1). In addition, we noticed that this ratio significantly increased in the case of mAb 3F11 to the C domain (Fig. 2A) - an effect that was not noticed for blood ACE in solution (not shown). Interestingly, in contrast to blood ACE, ZPHL/HHL ratio for recombinant ACE precipitated by mAb 1B8 decreased – for both soluble or membrane ACEs (Figure S1B and S1C), whereas an increase in ZPHL/HHL ratio precipitated by mAb 3F11 was not seen in the case of soluble recombinant ACE (Figure S1B). We consider these findings as an indication that two mAbs, 1B8 and 3F11, to the C domain are capable to induce conformational changes in ACE globule accompanying by the change of ZPHL/HHL ratio, however, the local conformation of the epitope for the particular mAb on the ACE surface, as well as capability for conformational changes induced by mAbs binding, are ACE-type specific.

**Figure 9:**
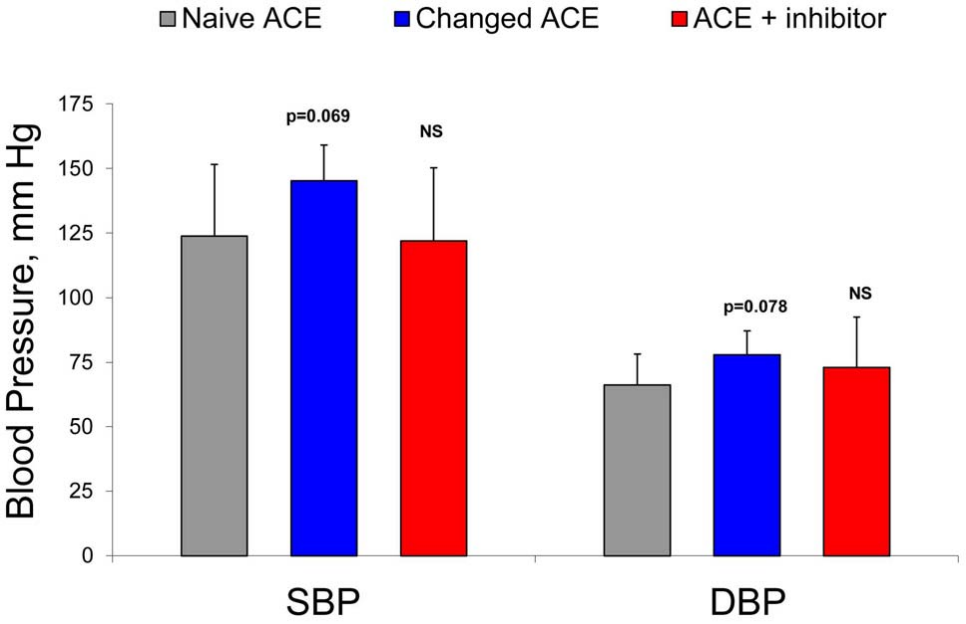
Blood pressure in patients with uremia due to ESRD. Blood pressure was measured in patients with ESRD before dialysis and before blood sampling. According to T test p values for the differences in Systolic Blood Pressure (SBP) and Diastolic Blood Pressure (DBP) between patients with conformationally naïve and conformationally changed ACE were close to the level of significance (p<0.05).

Figure 2, B and C, demonstrates that in the presence of high concentration (100 nM) of “short” ACE inhibitor enalaprilat the ZPHL/HHL ratio (after standard washing, 5×200 µl/well) slightly increased only in the case of mAb 1E10, which could be considered as an indication of some retention of enalaprilat inside ACE induced by this particular mAb. After extensive washing (10×200 µl/well) with Cl^–^free buffer, however, the ZPHL/HHL ratio in case of mAb 1E10 appeared to be equal to that for other mAbs and ACE in solution (data not shown), that is, enalaprilat bound to blood ACE was completely washed out before adding of ACE substrates.

Moreover, the fact that ZPHL/HHL ratio did not increased for ACE precipitated by mAbs 1G12/6A12 and by 1E10 in the presence of teprotide both after standard and intensive washings could be unequivocally considered as the evidence that there was no any retention of the inhibitor by these mAbs. So, the decrease of the precipitation of blood ACE activity by mAbs 1G12/6A12 and 1E10 in the presence of this inhibitor (Fig. 1) was due to a real decrease of these mAbs binding to blood ACE induced by teprotide binding within ACE active centers.

It is necessary to note that the effect of ACE inhibitors on mAbs binding to ACE depends both on the type of ACE inhibitor and the source of ACE. Thus, “long” ACE inhibitor teprotide similarly changed the pattern of mAbs binding to ACE from serum, seminal fluid or soluble recombinant human sACE, as well as truncated N or C domain expressed in CHO cells: the binding of mAbs 1G12 and 6A12 to the N domain dramatically decreased, 2–3 folds, in all cases, whereas the binding of mAb 1B3, directed to the C terminal part of the C domain, significantly increased (Figure S2 and S3). “Short” inhibitor enalaprilat showed more difference. This inhibitor similarly decreased the binding of mAbs 1E10 and 4E3 (directed to the C domain) to blood ACE, ACE from seminal fluid or soluble recombinant ACE. However, while the binding of mAbs 1G12 and 6A12 to blood ACE sharply increased in the presence of enalaprilat, the binding of mAb 1G2 to ACE from seminal fluid or soluble recombinant human ACE practically did not change and the amplitude of the effect of enalaprilat on mAb 6A12 binding was much less pronounced (Figures S2 and S3).

Therefore, data presented on Figures 2, S1-S3 demonstrated clearly, that the effects of ACE inhibitors on the mAbs binding, while ACE-type specific, confirmed one more time that the pattern of mAbs binding to ACE is the most sensitive marker for even subtle local conformational changes in ACE molecule.

The main goal of this study was to find putative conformational changes in ACE at disease/pathology by use of a conformational fingerprinting of blood ACE with a set of mAbs. Therefore processing of blood ACE for the future analysis could be a very important technological and methodological issue. By classical biochemical approach, an isolation of pure ACE from plasma/serum of patients with a disease seemed an ideal. However, when we performed purification of ACE from plasma or seminal fluid by lisinopril affinity chromatography we found that the pattern of mAbs binding (conformational fingerprint, i.e. local conformation of ACE) was rather different for purified ACE and the corresponding ACE in the content of biological fluid (Figure S4). Therefore we decided to use unprocessed serum/plasma as the source of naive blood ACE conformation from a given patient. It is important that ACE from serum, citrate plasma or heparinized plasma (but not EDTA-plasma) exhibited similar “conformational fingerprint” (data not shown), that is this particular blood processing did not influence ACE local conformation.

### Effect of Different Compounds on mAbs Binding to Blood ACE

Sensitivity of the pattern of binding of a set of mAbs to local conformational changes induced by glycan moiety [49] or by binding of ACE inhibitors ( [43] and this study) inspired us to hypothesize that the disease(s) characterized by high concentration of toxic compounds can be accompanied by the conformational changes in ACE in the blood. Therefore we performed screening of several compounds for their effect on mAbs binding to ACE.

Somatic ACE molecule contains 6 paired cysteine residues (Cys) forming S-S bridges [60]- Cys128-Cys136, Cys330-Cys348 and Cys526-Cys528 in the N domain, as well as Cys728-Cys734, Cys928-Cys946 and Cys1114-Cys1126 in the C domain, and two unpaired cysteines, Cys 474 and Cys 1072, which theoretically can participate in the creation of homo- or hetero- ACE dimers *via* disulfide bridges. Therefore, at first we tested compounds that can affect the disulfide bridge formation, dithiothreitol (DTT) and reduced glutathione (GSH).

Figure S5 demonstrates the effects of DTT and GSH on blood ACE conformational fingerprint. The reduction of S-S bridges by both reagents (Fig. S5, A and B) resulted in rather similar changes in mAbs binding pattern: the most significant effect was observed with mAbs 1G12 and i2H5 to the N domain and mAb 1B3 to the C domain which binding to ACE significantly increased as a result of the treatment. Epitopes for mAbs 1G12 and i2H5 contain the disulfide bridge Cys516-Cys528, therefore we can conclude that the reduction of this bridge resulted in the effect observed. The epitope for mAb 1B3 does not contain any S-S bridge, but the bridge Cys728-Cys734 is located nearby. The binding of mAb 3F11 to the C domain also increased after GSH (not DTT) treatment, its epitope containing Cys1114-1126; mAb BB9 slightly increased and mAb 1E10 decreased the binding to ACE due to GSH treatment, however, epitopes for these mAb do not contain any S-S bridge. Other mAbs, 3G8 (no S-S bridges in the epitope) and 5F1 (Cys330-Cys348 in the epitope) to the N domain, 1B8 and 3F10 (Cys728-Cys734 in epitopes) and 2H9 (no S-S bridges in the epitope) to the C domain decreased the binding with blood ACE due to DTT treatment. Similar but less pronounced effects of DTT and GSH on mAbs binding to ACE we obtained with purified ACE from seminal fluid (data not shown).

Another compound, 5,5′-dithiobis(2-dinitrobenzoic acid), capable to interact with free Cys residues, induced the only change in mAbs binding pattern – the 40%-decrease of the binding of mAb 1B3 (data not shown), epitope of which is localized on the C-terminal end of the C domain and contains free Cys 1072 [27].

Thus, these experiments demonstrated that the state of Cys residues and S-S bridges can play a remarkable role in the conformational fingerprint of ACE. It is known that the hemolysis of erythrocytes causes liberation of intracellular GSH to plasma thus increasing both GSH and GSSH (oxidized glutathione, due to auto-oxidation of GSH in the extracellular medium) plasma concentrations [61]–[62]. So, we tested the effect of hemolysis on mAbs binding to blood ACE and showed that the binding of three mAbs to the N domain, 1G12, 6A12 and i2H5 (epitopes of all three contain Cys516-Cys528 bridge), remarkably increased (Fig. S5, C). So, we unequivocally demonstrated that the compounds present in blood can directly influence the conformation of ACE globule as seen by ACE conformational fingerprint. It is worth noting, as a methodological remark, that plasma/serum should not be hemolysed prior this fingerprinting.

### Conformational Fingerprinting of Blood ACE in Uremic Patients

Uremia is a pathology characterized by a high content of different toxic compounds [51] due to retention of these compounds and a deficient renal clearance caused by kidneys disease. Patients suffering from chronic renal failure are exposed to increased oxidative stress generated by uremic toxins, further exacerbated by HD, chronic inflammatory state, etc. [63]–[65]. Specifically, GSH was reported to be significantly elevated in the ESRD and HD patients [66]–[67]. Therefore, we chose uremic state in HD ESRD patients to be checked for the effect both on the activity and on the conformation of blood ACE.

Figures 3 demonstrates an example of this approach (ACE phenotyping) when patient plasma/serum was tested for 1) ACE activity with two substrates, ZPHL and HHL; 2) the presence of ACE inhibitors in patient’s blood using enzymatic assay – ZPHL/HHL ratio [54] and more sensitive antibody-based assay –1G12/9B9 ratio [43]; 3) concentration of ACE protein (using immuno-capture assay with mAb 9B9 [37]); 4) conformational control of the most labile region of ACE – the region of the epitope for mAb 1G12 overlapping with epitopes for mAbs 6A12 and i2H5 [43].

Figure 3A shows ACE activity in 20 ESRD HD patients, whose blood was taken before HD and in 7 healthy volunteers. Five ESRD patients (out of 20) demonstrated an increased ACE activity in their blood which corroborate with several publications that reported increased ACE activity in uremic patients [21]–[23]. Figure 3B demonstrates the ratio of the rates of hydrolysis of two substrates (ZPHL/HHL ratio) for all these patients. This parameter [54] is very stable in a normal population, 100±3.0%, while ACE activity itself measured with any substrate deviates very significantly, 100±30%, [16], [37]. It is clearly seen that ZPHL/HHL ratio was significantly increased in 4 (out of 20, numbers 2, 4, 12 and 15) ESRD patients. This result indicates the presence of ACE inhibitor in the blood of these patients at the time of blood sampling [54] and confirms the necessity of the objective testing of presence of ACE inhibitors in the patient’s blood in all clinical trials which can include treatment with ACE inhibitors.

We performed also antibody-based assay and estimated the precipitation of blood ACE from these patients by two mAbs to ACE – mAb 9B9 (Figure 3E) which binding to blood ACE is not affected by the presence of ACE inhibitors and corresponds to the ACE protein in the blood [37], and mAb 1G12 (Figure 3D), which binding to blood ACE remarkably increased (3-5-fold) in the presence of common ACE inhibitors [43]. We confirmed that both the binding of mAb 1G12 (Figure 3D) and, more important, 1G12/9B9 ratio (Figure 3C) increased dramatically in those 4 patients (numbers 2, 4, 12 and 15) that were detected as having ACE inhibitors in their blood with the help of ZPHL/HHL ratio (Figure 3B).

This approach, however, allowed us to discover another four ESRD patients (out of 20, numbers 7, 11, 13 and 16 - boxed in Figures 3B and C), whose 1G12/9B9 ratio increased while ZPHL/HHL ratio was absolutely normal. So, we could consider blood ACE (at least) in these patients as having local conformational changes in 1G12 epitope. It is important that these changes could not be attributed to the presence of ACE inhibitors (as common drugs at hypertension) in the blood of these patients but should be considered as conformational changes of ACE surface caused by some toxic compounds due to uremia.

We tested the binding of the set of mAbs to ACE from plasma of those ESRD patients (numbers 7, 11, 13 and 16) compared to that for healthy persons and found that besides the increase of binding of mAb 1G12 (Figure 3, C and D) only mAb 1B3 to the C domain (2 mAbs out of 15) bound better to ACE from the blood of ESRD patients (Figure 4A). One of the reasons for such an increase could be an effect of putative toxic compounds on the disulfide bridge Cys516-Cys528 in the epitope for mAb 1G12 and on the disulfide bridge Cys728-Cys734 close to epitope for mAb 1B3 and/or on unpaired Cys1072 directly in the epitope for mAb 1B3 (Figure 4B) similar to the effects obtained after hemolysis or after ACE treatment with DTT, GSH and DTNB (Fig. S5). It should be pointed out, however, that the principal difference between the abovementioned effects and uremia effect exists: the binding of mAbs 6A12 and i2H5 (having overlapping epitopes with epitope for mAb 1G12) with ACE from the blood of ESRD patients changed only very slightly (Fig. 4A) compared with the significant increase of the binding of mAb i2H5 after GSH and DTT treatments and increase of the binding of all three mAbs, 1G12, 6A12 and i2H5, due to hemolysis (Fig. S5).

The next logical step was an analysis of the effect of the presence of “short” (enalaprilat) and “long” (teprotide) inhibitors on the local conformation of blood ACE (expressed as 1G12/9B9 ratio) from ESRD patients versus that of healthy volunteers. Figure 5 clearly demonstrates that both healthy donors (mean value for 33 persons) and ESRD patients (numbers 1, 3, 9, 14, 17 and 18) having low 1G12/9B9 ratio responded to enalaprilat in a common way, i.e. by a significant increase of 1G12/9B9 ratio. However, the response of ESRD patients with high 1G12/9B9 ratio (patients 2, 7, 13, 16, 2 and 12, the last two having ACE inhibitors in the blood as shown above) did not show significant response (if any) to the presence of enalaprilat (Fig. 5, A and B). It seems that in the blood of these ESRD patients ACE possesses the local conformation with most accessible epitope for mAb 1G12, otherwise partially buried/hidden in ACE from healthy persons. The action of enalaprilat on ACE, besides activity inhibition, is the increase of accessibility of this epitope to the mAb which appeared to be impossible for these ESRD patients (including persons having ACE inhibitors in the blood) as 1G12 epitope on ACE from their blood is already fully accessible.

A rather different situation was observed with teprotide: the effect of the inhibitor on mAbs binding with ACE from uremic patients in both groups, with low and high 1G12/9B9 ratios, was less pronounced than that for healthy persons (Fig. 5, C and D). However, the response to teprotide appeared to be individual, as in both groups there were patients with very weak (numbers 1, 3, and 13) and almost normal (number 7) decrease of mAb 1G12 binding to ACE in the presence of teprotide. The binding of mAb 1G12 to ACE from the blood of patients (numbers 2 and 12) having ACE inhibitors did not decrease at all in the presence of teprotide, indicating that teprotide could not reverse the effect of ACE inhibitors in the blood.

The fact that ACE inhibitors became unable to induce conformational changes on the surface of ACE from blood from some ESRD patients (Figures 3,4,5) put the question about ACE response to these inhibitors regarding inhibition of the enzyme activity. At first we tested an effect of anti-catalytic mAbs to ACE on the rate of the hydrolysis of substrates ZPHL (with mAbs 3A5 and 12H5 to the N domain) and HHL (with mAb 4E3 to the C domain) by ACE from uremic plasma and several samples of plasma from healthy donors. Figure 6 demonstrates that at least three (out of 4) ESRD patients with elevated 1G12/9B9 ratio exhibited significantly diminished efficacy of anti-catalytic action of mAbs 3A5 and i2H5 to the N domain and two ESRD patients exhibited diminished efficacy of mAb 4E3 as anti-catalytic mAbs to the C domain (Fig. 6).

The most important results were obtained in the experiments with inhibition of ACE from the blood of ESRD patients compared with that from healthy donors with both ZPHL and angiotensin I as substrates (Fig. 7). First of all, the activity of ACE in the blood of some ESRD patients, especially with high 1G12/9B9 ratio, in the reaction of the hydrolysis of angiotensin I appeared to be much higher (up to 5-fold) than the activity of ACE in the blood of healthy donors (Fig. 7E). This result couldn’t be explained by higher level of ACE in uremic plasma, as we did not find any increase in ACE binding with mAb 9B9 (Fig. 3E). Besides, the activity of these patients with ZPHL as a substrate was similar to that of healthy donors (Fig. 3A).

Figure 7A and B shows that patients with high 1G12/9B9 ratio are slightly less susceptible to inhibition of the hydrolysis of ZPHL by “short” ACE inhibitor enalaprilat (Fig. 7 A) and even less susceptible to the inhibition of the hydrolysis of angiotensin I by this inhibitor (Fig. 7B). “Long” ACE inhibitor teprotide distinguished patients with low and high 1G12/9B9 ratios even more effectively and less dependently on the structure of the substrate: blood ACE from patients with high 1G12/9B9 ratio was inhibited by 1 µM teprotide only slightly with both ZPHL and angiotensin I as substrates (Fig. 7, C and D). Moreover, in the latter case inhibition could be considered as negligible. To prove it true, we diluted plasma from ESRD patients 5-fold to make it equal to normal plasma by ACE activity with angiotensin I as a substrate. However, even at this case ACE from blood of ESRD patients with high 1G12/9B9 ratio failed to be inhibited with the same effectiveness as ACE in normal blood (data not shown). It is worth noting that we did not find any dependence of these effects on the age or gender of patients.

It is known, that cardiovascular events are the leading cause of death in adult ESRD patients on maintenance HD with annual mortality rate about 20% [68]–[69]. The current recommendation is to employ a renin-angiotensin system (RAS)-blocking agents, ACE inhibitors in particular [70]–[71]. However, the majority of cardiovascular primary and secondary prevention clinical trials have excluded patients with advanced renal insufficiency [72]–[73]. Therefore, it is less known whether the results of clinical trials could be also applied to those patients, who are on maintenance HD with a mixture of uremic toxic compounds in their serum. In a retrospective study, patients with renal failure on HD treated with ACE inhibitors had a lower mortality rate as compared to those who were not treated [74]–[75]. However, observational analyses alone are not sufficient to assess the efficacy and safety of ACE inhibitor use in this setting [72]. In randomized controlled Fosinopril Study in Dialysis (FOSIDIAL) no significant benefit for Fosinopril was observed in the intent-to-treat analysis after adjusting for independent predictors of cardiovascular events [72]. Similar conclusion, but with different ACE inhibitors, was made in HEMO study representing randomized clinical trial of HD patients regarding flux and dose of dialysis [72]. This study fail to show an association of ACE inhibitors use with less cardiovascular morbidity and mortality of HD patients, while beneficial effect of ACE inhibitors on CVE risk reduction in general populations was repeatedly and clearly demonstrated [76]. In another study, in children with chronic renal failure, the use of ACE inhibitors in ESRD patients failed to decrease plasma angiotensin II level at all [77].

Different mechanisms underlying therapy resistance to ACE inhibitors have been suggesting so far: ACE gene polymorphism [78]–[79], the extent of renal damage prior to ACE inhibition [80], different drug clearance in responders and non-responders [81]. We can hypothesize, however, on the base of obtained results, that in uremic state the response to ACE inhibitors could be markedly attenuated by uremic toxicity and possible reason might be conformational changes in ACE molecule, induced by elevated levels of toxic substances in the uremic serum.

Therefore, we can consider that simple and technological control of ACE conformation - determination of 1G12/9B9 ratio for blood ACE in the conjunction with the simultaneous determination of ZPHL/HHL ratio, altogether with determination of ACE activity with angiotensin I as a substrate, can identify subpopulation of patients (at least ESRD patients) less sensitive to ACE inhibitor therapy and who, therefore, either should be treated with these drugs more aggressively or use alternative approach to RAS blockade.

In order to estimate the frequency of conformationally changed blood ACE in a wide population we performed ACE phenotyping and determined these parameters, 1G12/9B9 and ZPHL/HHL ratios, in 63 unrelated patients and in 48 healthy young blood donors. The result of the 1^st^ round of such ACE phenotyping (in 48 healthy young donors) is presented in Figure 8. ACE activity in this population (within 95%) differed 2.3-fold (Fig. 8A), and ACE protein in the blood in this population (according to mAb 9B9 binding) differed 2.2-fold (Fig. 8B), which showed high accuracy of the determination of ACE activity and protein and confirmed high diversity of ACE level in population [16], [37]. ZPHL/HHL ratio was a very stable parameter (Fig. 8C, 100±4.4%). In this population of 48 patients we found 3 patients (Fig. 8D, numbers 11, 13 and 18) with significantly elevated 1G12/9B9 ratio (with normal ZPHL/HHL ratio).

As a whole, we found 4 patients with elevated 1G12/9B9 ratio, while normal ZPHL/HHL ratio, from 20 ESRD patients (that is 20%); 5 patients of such characteristics from 63 patients without chronic kidney diseases (7.9%), while only 3 healthy donors out of 48 (6.3%) exhibited elevated 1G12/9B9 ratio. Thus, in a normal population the percentage of blood ACE with changed conformation is remarkably lower than that in chronic uremia in ESRD.

The level of 1G12/9B9 ratio reached 240–490% in the blood of ESRD patients from that for healthy controls, while for patients with unrelated diseases and for healthy persons this level did not exceed 230–240% for two persons and was only about 145–185% for other 6 persons with elevated 1G12/9B9 ratio (Fig. S6, F). It is important that those persons exhibiting higher 1G12/9B9 ratio (more than 200% from control) exhibited higher ACE activity towards angiotensin I as well (Fig. S6, E). The inhibition of ACE from the blood of healthy/unrelated patients with lower 1G12/9B9 ratio (145–185%) didn’t differ from that for control persons both by enalaprilat and teprotide with ZPHL and angiotensin I as substrates (Fig. S6, A–D). However, the inhibition of blood ACE activity for the patients with higher 1G12/9B9 ratio (230–240% from control) by both enalaprilat (Fig. S6, B) and teprotide (Fig. S6, D) appeared to be less effective than for normal ones with angiotensin I as a substrate, similar to that for ACE from the blood of ESRD patients.

Thus, elevated 1G12/9B9 ratio for blood ACE could be considered as marker for conformationally altered ACE, which inhibition by ACE inhibitors could be impaired in uremic state and, thus, could mark non-responders to ACE inhibitors.

What could be the reason for this particular region on the blood ACE molecule (epitopes for mAb 1G12/6A12) to be so sensitive to conformational changes induced by ACE inhibitors (Fig. 1), SH-reagents (Fig. S5), ACE purification (Fig. S4), uremia (Fig. 3,4,5, 7), whereas binding of these mAbs to recombinant human somatic ACE or individual domains was less influenced by these reagents? Figure S7 shows comparative effects of ACE inhibitor enalaprilat and fetal bovine and human sera on mAbs binding to soluble human recombinant sACE. Effect of enalaprilat on mAbs binding to recombinant ACE (Fig. S7B) was similar to human serum ACE (Fig. S7A) for all mAbs, except mAbs 1G12 and 6A12, which binding to recombinant ACE increased to less extent. Fetal bovine serum (10%) slightly increased the binding of these mAbs to recombinant ACE (Fig. S7C) which can indicate on the presence of endogenous ACE inhibitors in bovine serum that were previously found in plasma from different animals, including human [54, 82–84, and own unpublished data]. However, adding of 10% of human serum to recombinant ACE dramatically decreased the binding of 3 mAbs, 1G12, 6A12 and i2H5, directed to overlapping epitopes on the N domain of ACE (Fig. S7D). We can consider this fact as an indication that some component of human plasma (ACE-binding protein?) binds to the region of the overlapping epitopes for these mAbs on the N domain of recombinant ACE and decrease, therefore, the binding of mAbs. In this case, dramatic increase of mAbs 1G12 and 6A12 binding to blood ACE in the presence of commercial “short” ACE inhibitor, as a result of treatment with GSH or DTT or after action of toxic compounds in the blood of ESRD patients could be partially explained by a possible dissociation of this putative ACE-binding protein from its complex with the enzyme with simultaneous unmasking the epitopes for these mAbs. Several ACE-binding proteins (some of them with ACE-inhibiting properties) were suggested so far: 100 kD protein [85]–[86], 14 kD protein [87], but the nature of these proteins was not yet identified.

Finally we tried to link our findings with surrogate clinical end-point, such is blood pressure (BP) - Table 1. There was a clear (however, not statistically significant) trend towards differences in pre-dialysis BP in those patients, who demonstrate conformational changes of ACE, and other patients (Fig. 9). Systolic BP for the patients with conformationally changed ACE was 145.3±13.9 mm Hg versus 122.0±28.3 (p = 0,069) for other patients, while diastolic BP was 78.0±9.1 mm Hg versus 66.2±12.1, respectively (p = 0.078).

### Conclusions

We demonstrated that the pattern of precipitation of ACE activity by mAbs to ACE (conformational fingerprinting of ACE) is a sensitive parameter characterizing changes in the local ACE conformation. We have shown that this pattern of ACE binding by a set of mAbs was influenced significantly by the presence of ACE inhibitor, common inhibitor (tripeptide analog) and teprotide (nonapeptide) causing different effects. Conformational characteristics of ACE can be also changed by the action of compounds capable to interact with S-S bridges within protein globule or with free Cys residues. The conformational fingerprinting of ACE together with technological enzymatic test – the determination of relative rates of the hydrolysis of two substrates, ZPHL and HHL, appeared to have a potential for the selection of a group of patients among ESRD patients which are not sensitive enough for a common treatment by ACE inhibitors. ACE in the blood of these patients is characterized by elevated 1G12/9B9 ratio (relative binding of two mAbs to different epitopes on the surface of ACE N domain) and elevated activity with angiotensin I as a substrate, while normal activity with ZPHL and normal ZPHL/HHL ratio. ACE activity in the blood of these patients is worse inhibited both by common ACE inhibitor and teprotide.

These findings indicate that conformational fingerprinting of ACE can help to indicate the occurrence of the changes of the protein due to disease and may be considered as an approach for the finding of risk factors at pathology.

Despite obvious limitation of our study that was not designed to evaluate BP in this high risk group of patients, our results in the group of long-term survivals on maintenance HD with long time exposure to chronic uremia reveal that conformational changes of ACE might participate in the mechanism of adverse clinical outcomes in ESRD patients.

## Supporting Information

Figure S1 **ZPHL/HHL ratio for different ACEs precipitated by mAbs to ACE.** Pooled citrated plasma diluted 1/5 with PBS was equilibrated by ACE activity with soluble or membrane form of human recombinant ACE (5 mU/ml with HHL as a substrate) and incubated with microtiter plate coated with 16 different anti-ACE mAbs via goat-anti-mouse IgG bridge as in Figure 1. Precipitation of ACE activity by these mAbs from different ACE sources was expressed as a ratio of precipitated ACE activity with substrate ZPHL to that with substrate HHL (Danilov et al. 2008). Pooled citrated plasma from 30 healthy individuals Culture medium from CHO cells transfected with human recombinant ACE (clone 2C2 -Balyasnikova et al. 1999) –soluble WT ACE. Lysate from CHO cells transfected with human recombinant ACE (clone 2C2 -Balyasnikova et al. 1999) – membrane form of WT ACE. The red color of the bars shows higher ZPHL/HHL ratio – more than 20% (and yellow – lower) obtained for ACE precipitated by corresponding mAb compared to that in solution. * – p<0.05 in comparison with ZPHL/HHL ratio of a given ACE in solution.
(TIF)

Figure S2 **Effect of ACE inhibitors on mAbs binding to plasma and seminal fluid ACE.** **A**–**B**, **D**–**E.** The effect of enalaprilat (100 nM, **A** and **B**) or teprotide (1 µM, **D** and **E**) on mAbs binding to seminal fluid ACE (**B** and **E**) in comparison with plasma ACE (**A** and **D**) was determined using plate precipitation assay with ZPHL as a substrate as in Figure 1. The red color of the bars shows higher mAbs binding – more than 20% (and yellow – lower) obtained in the presence of inhibitors. *, p<0.05 in comparison with corresponding values obtained without inhibitor. **C**–**F**. The ratio of the effects of the inhibitor (enalaprilat, C, and teprotide, F) on mAbs binding with seminal fluid ACE to that for plasma ACE. The red color of the bars shows higher effects ratio – more than 20% (and yellow – lower) in comparison with the mean value for the whole mAbs set. All other terms and conditions are as in Fig. 1. Data are mean ± SD of 3–8 independent experiments (each in duplicates). * - p<0.05 in comparison with mean value for plasma ACE.
(TIF)

Figure S3 **Effect of ACE inhibitors on mAbs binding to soluble recombinant two-domain ACE (WTΔ) and individual truncated domains.** The effect of enalaprilat (100 nM, **A**–**C**) or teprotide (1 µM, **D**–**F**) on mAbs binding to soluble recombinant ACEs expressed in CHO cells was determined using plate precipitation assay with Z-Phe-His-Leu (ZPHL) as a substrate as in Figure 1. **A, D**. Soluble truncated human recombinant two-domain **s**ACE: 1–1230 WTΔ (Wei et al. 1991). **B, E**. Truncated recombinant N domain: 1–629 (Balyasnikova et al. 2003). **C, F**. Truncated recombinant C domain: 1–4, 613–1203 (Balyasnikova et al. 2005). All other terms and conditions-as in Figure 1. Data are mean ± SD of 3–4 independent experiments (each in duplicates). * - p<0.05 in comparison with values for samples without ACE inhibitors.
(TIF)

Figure S4 **Effect of ACE purification on the local conformation of ACE.** The effect of the purification of ACE from plasma and from seminal fluid by affinity chromatography on the lisinopril-Sepharose on the conformational fingerprint of ACE was assessed using 16 mAbs to different epitopes on the N and C domains of ACE. Pooled citrated plasma diluted 1/5 with PBS and seminal fluid (diluted 1/150) were equilibrated by activity with corresponding purified ACEs (approximately to 5 mU/ml). Precipitation of ACE activity by the set of mAbs was performed as in Figure 1 and expressed as a ratio of precipitated ACE activity from pure ACE to that from the corresponding source (plasma or seminal fluid). Plasma ACE. Seminal fluid ACE. All other terms and conditions are as in Figure 1. Red columns shows bigger (yellow – lower) precipitation of pure ACE activity (more than 20%) that that of ACE from biological fluid. Data are mean ± SD of 3 independent experiments (each in duplicates). * - p<0.05 in comparison with values for ACE from biological fluids.
(TIF)

Figure S5 **Effect of different compounds on mAbs binding to blood ACE. A, B**. Reduced glutathione and dithiotreitol at the indicated concentrations were incubated with pooled citrated plasma from 33 healthy volunteers (diluted 5-fold in PBS) for half an hour at 25°C before plate precipitation assay. **C**. Plasma sample was hemolysed by adding water and vigorous stirring and incubated for three days before the assay. Data were expressed as a ratio of precipitated ACE activity from plasma sample with tested compound/hemolysis to that without treatment. Red columns shows bigger (yellow – lower) precipitation of treated ACE activity (more than 20%) that that of ACE without treatment.Mean (+/− SD) from three independent experiments (each in duplicates). * - p<0.05 in comparison with mean values for samples without treatment.
(TIF)

Figure S6 **Effect of ACE inhibitors on the blood ACE activity of healthy donors/unrelated patients with normal** ***versus*** **high 1G12/9B9 ratio. A**–**D** Citrated plasma samples of unrelated patients and healthy donors with normal (patients #2 healthy and #19 unrelated), elevated (patients ##11 and 18 healthy; ##20, 32, 46, and 54 - unrelated) and high (patients #13 healthy and #21 unrelated) 1G12/9B9 ratio were incubated with “short” ACE inhibitor enalaprilat (100 nM, **A** and **B**) and “long” ACE inhibitor teprotide (1 µM, **C** and **D**) for 1 hour as in Fig. 7. Data are presented as a residual ACE activity determined with “short” substrate ZPHL (0.5 mM, **A** and **C**) and “long” substrate angiotensin I (0.3 mM, **B** and **D**). **E**. The ratio of the rates of the hydrolysis of angiotensin I and ZPHL (angiotensin I/ZPHL ratio) for corresponding samples. **F**. mAb 1G12/9B9 binding ratio for corresponding samples expressed as % from the mean value for controls. Grey bars – inhibition of ACE activity (A–D) or parameters measured in E–F in tested samples was not differed from that for healthy patients with low 1G12/9B9 ratio (controls). Red bars –measured parameters were statistically higher that in healthy patients with low (normal) 1G12/9B9 ratio *, p<0.05 in comparison with mean value for healthy patients. Data are mean ± SD from 3 independent experiments (each in duplicates).
(TIF)

Figure S7 **Effect of ACE inhibitor and bovine/human sera on mAbs binding to ACEs** The effect of enalaprilat (100 nM) on mAbs binding to plasma ACE (**A**) or enalaprilat (100 nM)- (**B**) or Fetal Bovine Serum - FBS, 10% (**C**) or Human Serum −10% (**D**) on mAbs binding to soluble recombinant sACE was determined using plate precipitation assay with ZPHL as a substrate as in Figure 1. Human serum after incubation with enalaprilat **B–D.** Soluble truncated human recombinant two-domain **s**ACE: 1–1230 WTΔ (Wei et al. 1991), after incubation with enalaprilat (**B**), FBS (**C**) and Human Serum (**D**) for 1 hour. All other terms and conditions-as in Figure 1. Data are mean ± SD of 3–4 independent experiments (each in duplicates). * - p<0.05 in comparison with values for samples without ACE inhibitors.
(TIF)
