# Supplementary figures and images for: Conformational Changes of Blood ACE in Chronic Uremia
Source: PLoS One. 2012 Nov 16;7(11):e49290. doi: 10.1371/journal.pone.0049290 (PMC3500299; doi:10.1371/journal.pone.0049290)

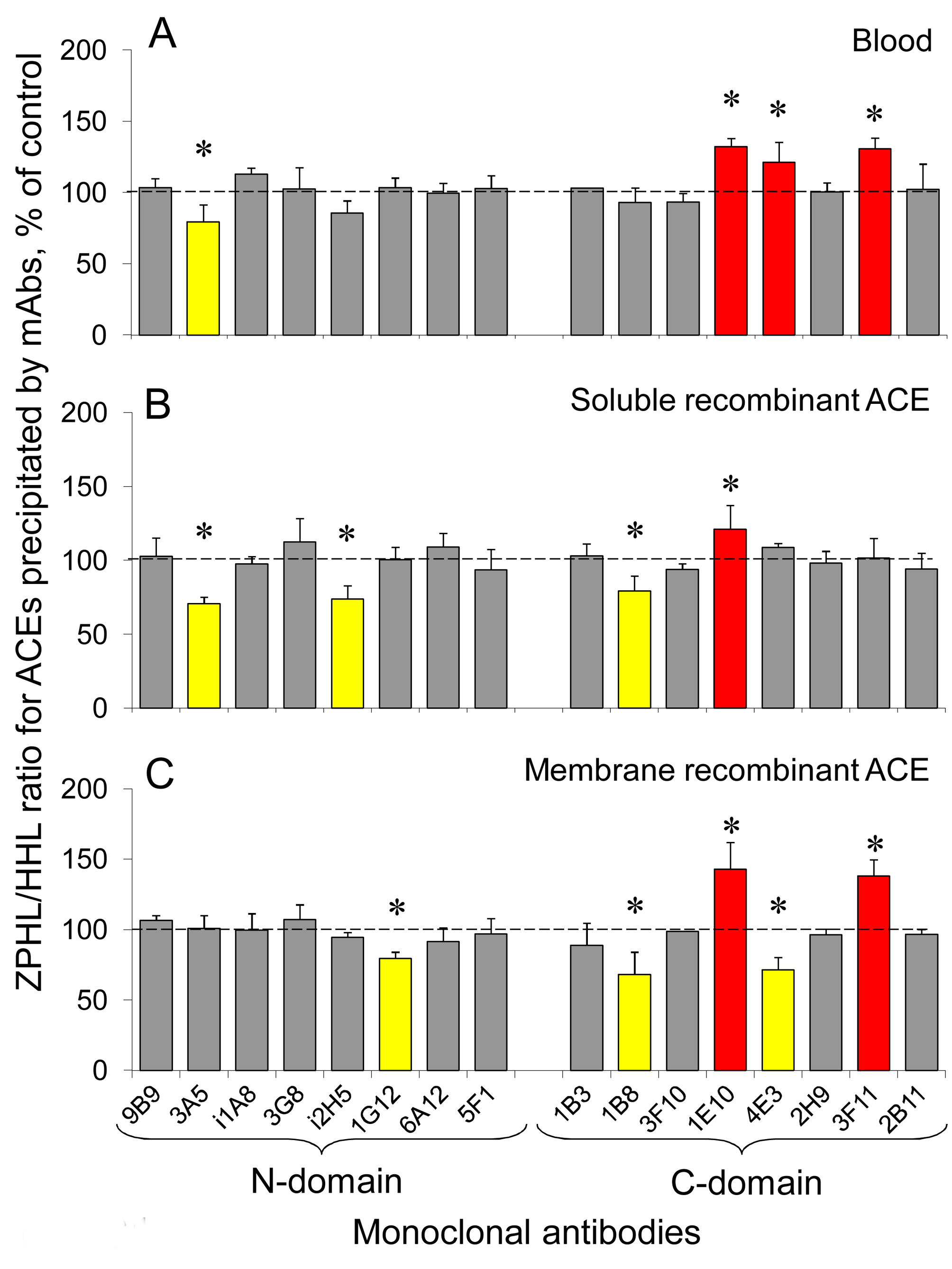

Supplement: Figure S1 — ZPHL/HHL ratio for different ACEs precipitated by mAbs to ACE. Pooled citrated plasma diluted 1/5 with PBS was equilibrated by ACE activity with soluble or membrane form of human recombinant ACE (5 mU/ml with HHL as a substrate) and incubated with microtiter plate coated with 16 different anti-ACE mAbs via goat-anti-mouse IgG bridge as in Figure 1. Precipitation of ACE activity by these mAbs from different ACE sources was expressed as a ratio of precipitated ACE activity with substrate ZPHL to that with substrate HHL (Danilov et al. 2008). Pooled citrated plasma from 30 healthy individuals Culture medium from CHO cells transfected with human recombinant ACE (clone 2C2 -Balyasnikova et al. 1999) –soluble WT ACE. Lysate from CHO cells transfected with human recombinant ACE (clone 2C2 -Balyasnikova et al. 1999) – membrane form of WT ACE. The red color of the bars shows higher ZPHL/HHL ratio – more than 20% (and yellow – lower) obtained for ACE precipitated by corresponding mAb compared to that in solution. * – p<0.05 in comparison with ZPHL/HHL ratio of a given ACE in solution. (TIF) [file pone.0049290.s001.tif]

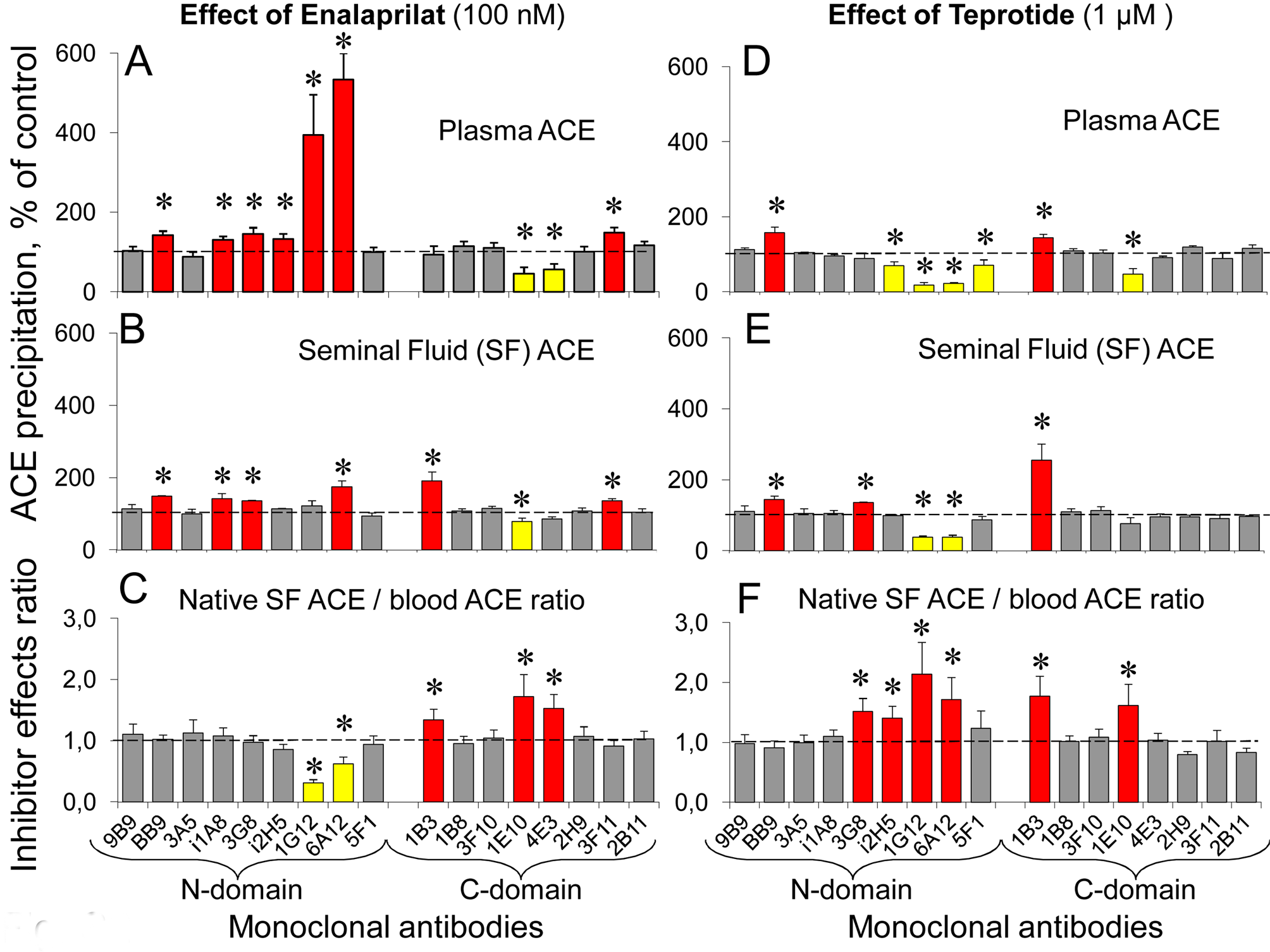

Supplement: Figure S2 — Effect of ACE inhibitors on mAbs binding to plasma and seminal fluid ACE. A–B, D–E. The effect of enalaprilat (100 nM, A and B) or teprotide (1 µM, D and E) on mAbs binding to seminal fluid ACE (B and E) in comparison with plasma ACE (A and D) was determined using plate precipitation assay with ZPHL as a substrate as in Figure 1. The red color of the bars shows higher mAbs binding – more than 20% (and yellow – lower) obtained in the presence of inhibitors. *, p<0.05 in comparison with corresponding values obtained without inhibitor. C–F. The ratio of the effects of the inhibitor (enalaprilat, C, and teprotide, F) on mAbs binding with seminal fluid ACE to that for plasma ACE. The red color of the bars shows higher effects ratio – more than 20% (and yellow – lower) in comparison with the mean value for the whole mAbs set. All other terms and conditions are as in Fig. 1. Data are mean ± SD of 3–8 independent experiments (each in duplicates). * - p<0.05 in comparison with mean value for plasma ACE. (TIF) [file pone.0049290.s002.tif]

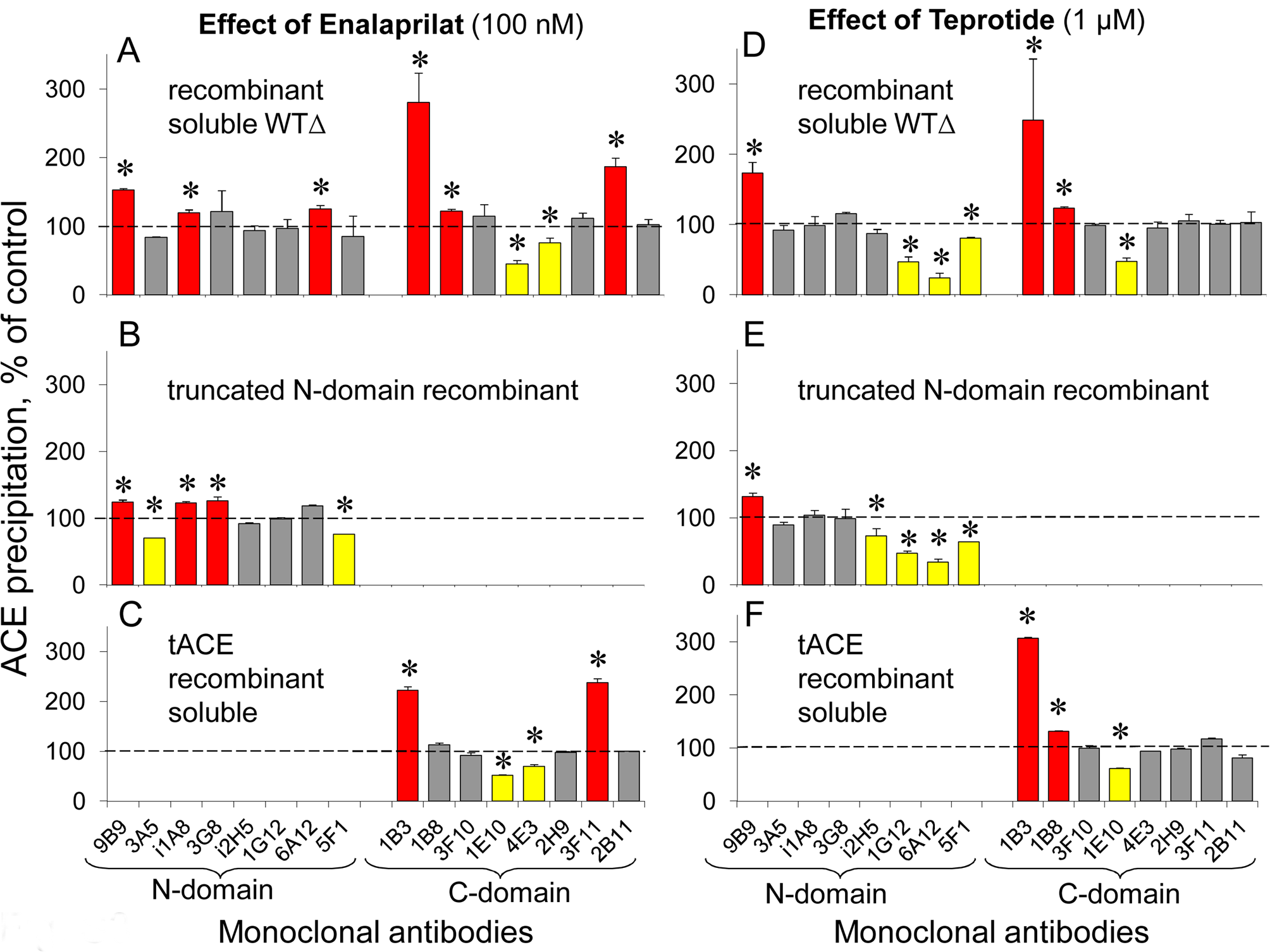

Supplement: Figure S3 — Effect of ACE inhibitors on mAbs binding to soluble recombinant two-domain ACE (WTΔ) and individual truncated domains. The effect of enalaprilat (100 nM, A–C) or teprotide (1 µM, D–F) on mAbs binding to soluble recombinant ACEs expressed in CHO cells was determined using plate precipitation assay with Z-Phe-His-Leu (ZPHL) as a substrate as in Figure 1. A, D. Soluble truncated human recombinant two-domain sACE: 1–1230 WTΔ (Wei et al. 1991). B, E. Truncated recombinant N domain: 1–629 (Balyasnikova et al. 2003). C, F. Truncated recombinant C domain: 1–4, 613–1203 (Balyasnikova et al. 2005). All other terms and conditions-as in Figure 1. Data are mean ± SD of 3–4 independent experiments (each in duplicates). * - p<0.05 in comparison with values for samples without ACE inhibitors. (TIF) [file pone.0049290.s003.tif]

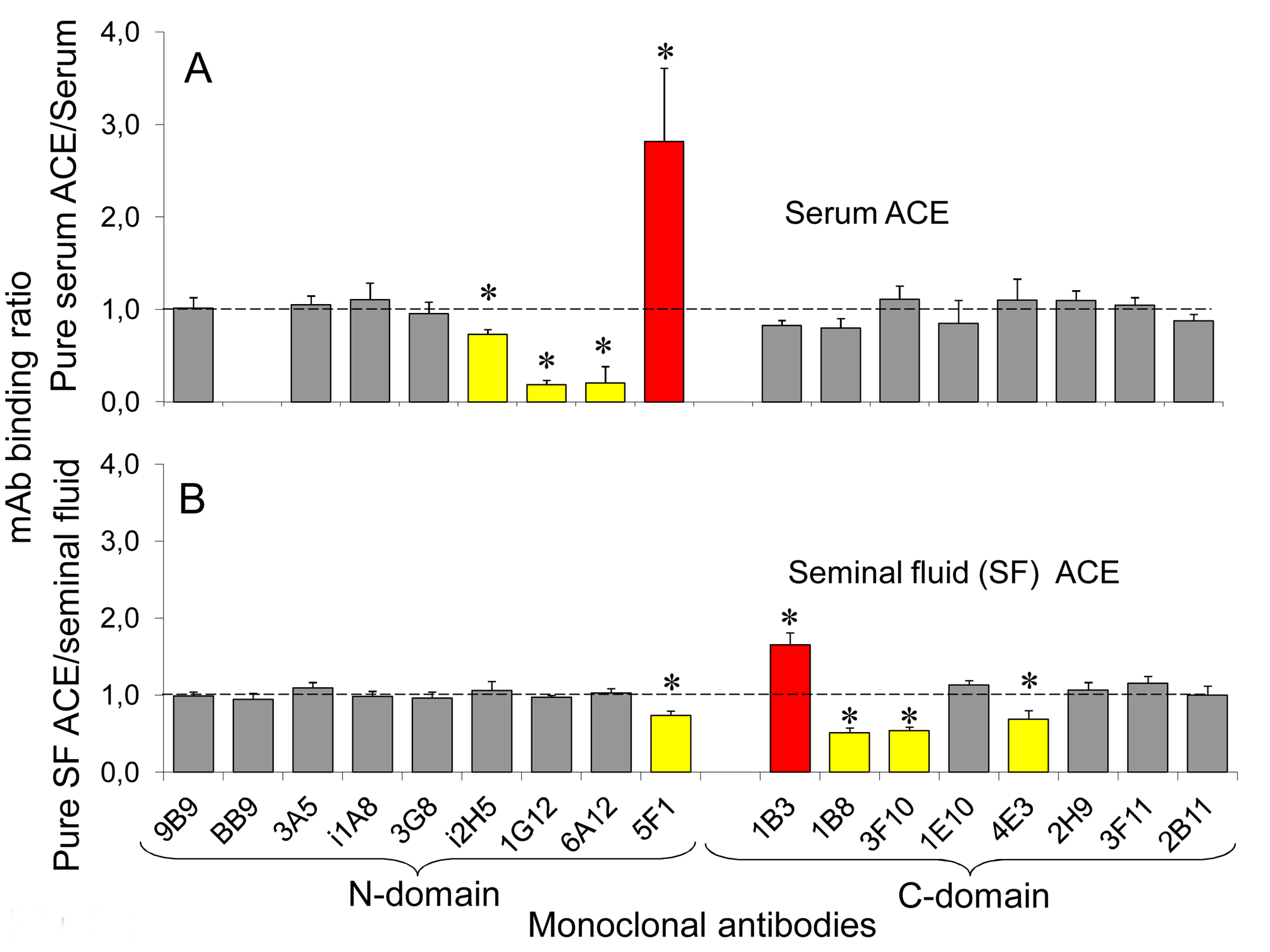

Supplement: Figure S4 — Effect of ACE purification on the local conformation of ACE. The effect of the purification of ACE from plasma and from seminal fluid by affinity chromatography on the lisinopril-Sepharose on the conformational fingerprint of ACE was assessed using 16 mAbs to different epitopes on the N and C domains of ACE. Pooled citrated plasma diluted 1/5 with PBS and seminal fluid (diluted 1/150) were equilibrated by activity with corresponding purified ACEs (approximately to 5 mU/ml). Precipitation of ACE activity by the set of mAbs was performed as in Figure 1 and expressed as a ratio of precipitated ACE activity from pure ACE to that from the corresponding source (plasma or seminal fluid). Plasma ACE. Seminal fluid ACE. All other terms and conditions are as in Figure 1. Red columns shows bigger (yellow – lower) precipitation of pure ACE activity (more than 20%) that that of ACE from biological fluid. Data are mean ± SD of 3 independent experiments (each in duplicates). * - p<0.05 in comparison with values for ACE from biological fluids. (TIF) [file pone.0049290.s004.tif]

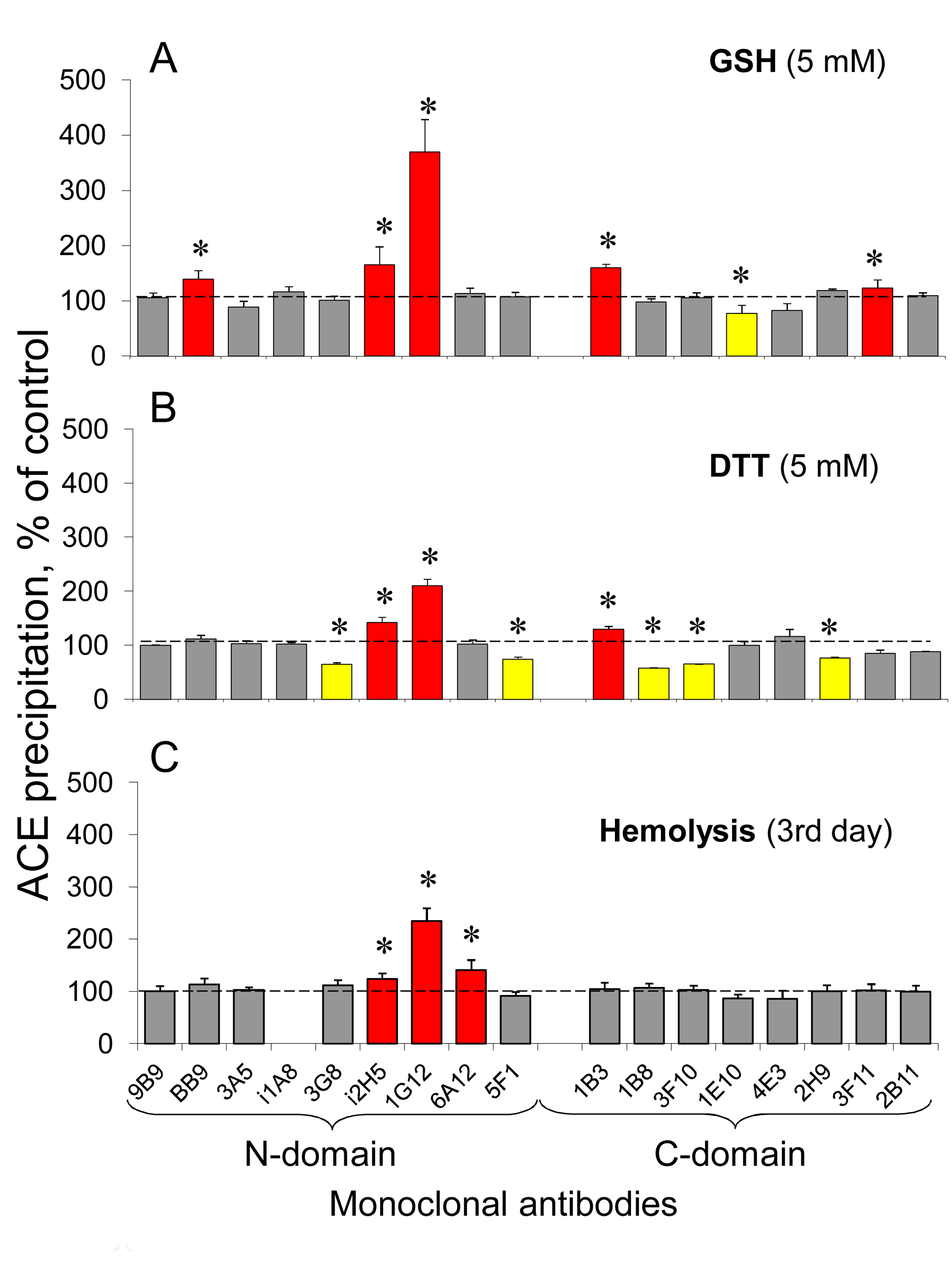

Supplement: Figure S5 — Effect of different compounds on mAbs binding to blood ACE. A, B. Reduced glutathione and dithiotreitol at the indicated concentrations were incubated with pooled citrated plasma from 33 healthy volunteers (diluted 5-fold in PBS) for half an hour at 25°C before plate precipitation assay. C. Plasma sample was hemolysed by adding water and vigorous stirring and incubated for three days before the assay. Data were expressed as a ratio of precipitated ACE activity from plasma sample with tested compound/hemolysis to that without treatment. Red columns shows bigger (yellow – lower) precipitation of treated ACE activity (more than 20%) that that of ACE without treatment.Mean (+/− SD) from three independent experiments (each in duplicates). * - p<0.05 in comparison with mean values for samples without treatment. (TIF) [file pone.0049290.s005.tif]

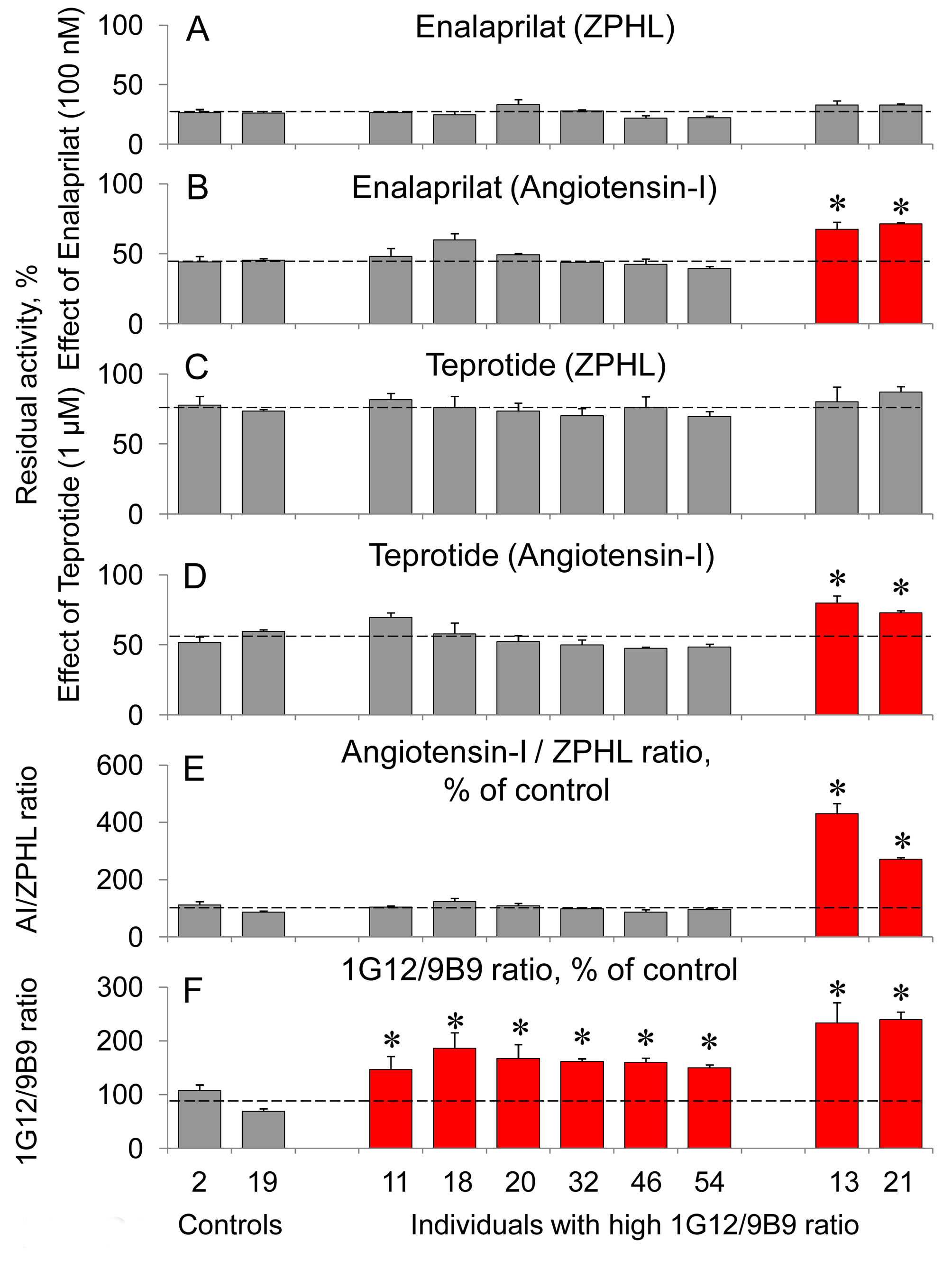

Supplement: Figure S6 — Effect of ACE inhibitors on the blood ACE activity of healthy donors/unrelated patients with normal versus high 1G12/9B9 ratio. A–D Citrated plasma samples of unrelated patients and healthy donors with normal (patients #2 healthy and #19 unrelated), elevated (patients ##11 and 18 healthy; ##20, 32, 46, and 54 - unrelated) and high (patients #13 healthy and #21 unrelated) 1G12/9B9 ratio were incubated with “short” ACE inhibitor enalaprilat (100 nM, A and B) and “long” ACE inhibitor teprotide (1 µM, C and D) for 1 hour as in Fig. 7. Data are presented as a residual ACE activity determined with “short” substrate ZPHL (0.5 mM, A and C) and “long” substrate angiotensin I (0.3 mM, B and D). E. The ratio of the rates of the hydrolysis of angiotensin I and ZPHL (angiotensin I/ZPHL ratio) for corresponding samples. F. mAb 1G12/9B9 binding ratio for corresponding samples expressed as % from the mean value for controls. Grey bars – inhibition of ACE activity (A–D) or parameters measured in E–F in tested samples was not differed from that for healthy patients with low 1G12/9B9 ratio (controls). Red bars –measured parameters were statistically higher that in healthy patients with low (normal) 1G12/9B9 ratio *, p<0.05 in comparison with mean value for healthy patients. Data are mean ± SD from 3 independent experiments (each in duplicates). (TIF) [file pone.0049290.s006.tif]

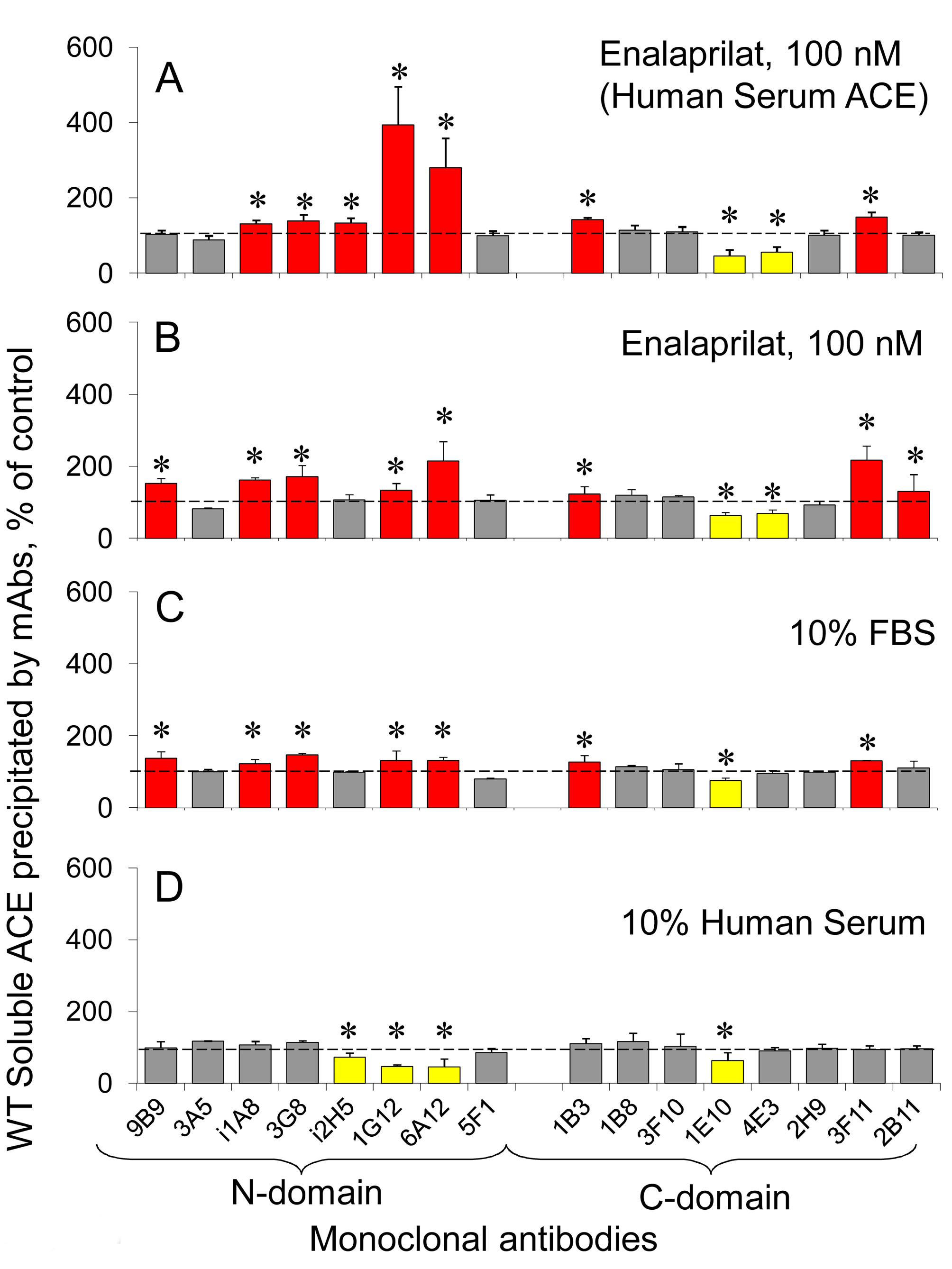

Supplement: Figure S7 — Effect of ACE inhibitor and bovine/human sera on mAbs binding to ACEs The effect of enalaprilat (100 nM) on mAbs binding to plasma ACE (A) or enalaprilat (100 nM)- (B) or Fetal Bovine Serum - FBS, 10% (C) or Human Serum −10% (D) on mAbs binding to soluble recombinant sACE was determined using plate precipitation assay with ZPHL as a substrate as in Figure 1. Human serum after incubation with enalaprilat B–D. Soluble truncated human recombinant two-domain sACE: 1–1230 WTΔ (Wei et al. 1991), after incubation with enalaprilat (B), FBS (C) and Human Serum (D) for 1 hour. All other terms and conditions-as in Figure 1. Data are mean ± SD of 3–4 independent experiments (each in duplicates). * - p<0.05 in comparison with values for samples without ACE inhibitors. (TIF) [file pone.0049290.s007.tif]
